# Biomarker-Guided Adaptive Trial Designs in Phase II and Phase III: A Methodological Review

**DOI:** 10.1371/journal.pone.0149803

**Published:** 2016-02-24

**Authors:** Miranta Antoniou, Andrea L Jorgensen, Ruwanthi Kolamunnage-Dona

**Affiliations:** 1 MRC North West Hub For Trials Methodology Research, Liverpool, United Kingdom; 2 Department of Biostatistics, Institute of Translational Medicine, University of Liverpool, L69 3GL, Liverpool, United Kingdom; The University of Queensland, AUSTRALIA

## Abstract

**Background:**

Personalized medicine is a growing area of research which aims to tailor the treatment given to a patient according to one or more personal characteristics. These characteristics can be demographic such as age or gender, or biological such as a genetic or other biomarker. Prior to utilizing a patient’s biomarker information in clinical practice, robust testing in terms of analytical validity, clinical validity and clinical utility is necessary. A number of clinical trial designs have been proposed for testing a biomarker’s clinical utility, including Phase II and Phase III clinical trials which aim to test the effectiveness of a biomarker-guided approach to treatment; these designs can be broadly classified into adaptive and non-adaptive. While adaptive designs allow planned modifications based on accumulating information during a trial, non-adaptive designs are typically simpler but less flexible.

**Methods and Findings:**

We have undertaken a comprehensive review of biomarker-guided adaptive trial designs proposed in the past decade. We have identified eight distinct biomarker-guided adaptive designs and nine variations from 107 studies. Substantial variability has been observed in terms of how trial designs are described and particularly in the terminology used by different authors. We have graphically displayed the current biomarker-guided adaptive trial designs and summarised the characteristics of each design.

**Conclusions:**

Our in-depth overview provides future researchers with clarity in definition, methodology and terminology for biomarker-guided adaptive trial designs.

## Introduction

The rapidly developing field of ‘personalized medicine’ [1], also known as ‘individualized medicine’, ‘stratified medicine’, or ‘precision medicine’ is allowing scientists to treat patients by providing them with a specific regimen according to their demographic or individualized genomic or biological characteristics, known as biomarkers [2]. The terms Personalized Medicine and Individualized Medicine often create confusion in literature as in reality, the objective of this approach is to identify demographic- or biomarker-defined subgroups. Thus, as it still remains a population and not an individualized approach, the terms Stratified or Precision medicine are often considered to be more accurate.

The Biomarkers Definitions Working Group defined a biomarker to be “a characteristic that is objectively measured and evaluated as an indicator of normal biological processes, pathogenic processes, or pharmacologic responses to a therapeutic intervention” [1, 3–6]. Biomarkers related to clinical outcome which are measured before treatment can be classified as either prognostic or predictive biomarkers. Prognostic biomarkers provide information regarding the likely progression of a disease without taking into account any specific treatment, whilst predictive biomarkers provide information about the patient’s outcome given a certain treatment, i.e. their likely response to the treatment [3, 6–33].

Prior to utilizing a patient’s biomarker information in clinical practice, it is necessary that they have been robustly tested in terms of analytical validity (this answers the question whether or not we should trust the results of a specific biomarker), clinical validity (the results obtained from the test should be related to other clinical information) and clinical utility (a particular biomarker should be useful in ameliorating patients’ health) [8, 12, 18, 24].

A number of Phase II and Phase III trial designs have been proposed for testing the clinical utility of predictive biomarkers and they can be broadly classified into adaptive and non-adaptive trial designs. As we enter the new era of personalized medicine, there is substantial need for novel trial designs which will (i) demonstrate cost benefits and minimize the required time to conclusive results despite an increase in the number of subjects needed for the trial; (ii) avoid erroneous conclusions and (iii) be more ethical by giving patients more effective treatments. Whereas non-adaptive trial designs often result in large and costly Phase III trials of long duration, adaptive designs are becoming increasingly attractive in the context of biomarker-directed therapies as they allow for additional flexibility during the course of the trial.

The current study aims to communicate the different biomarker-guided adaptive trial designs proposed in the literature so far, and to report on the potential advantages and weaknesses of each.

## Methods and Findings

**Fig 1:**
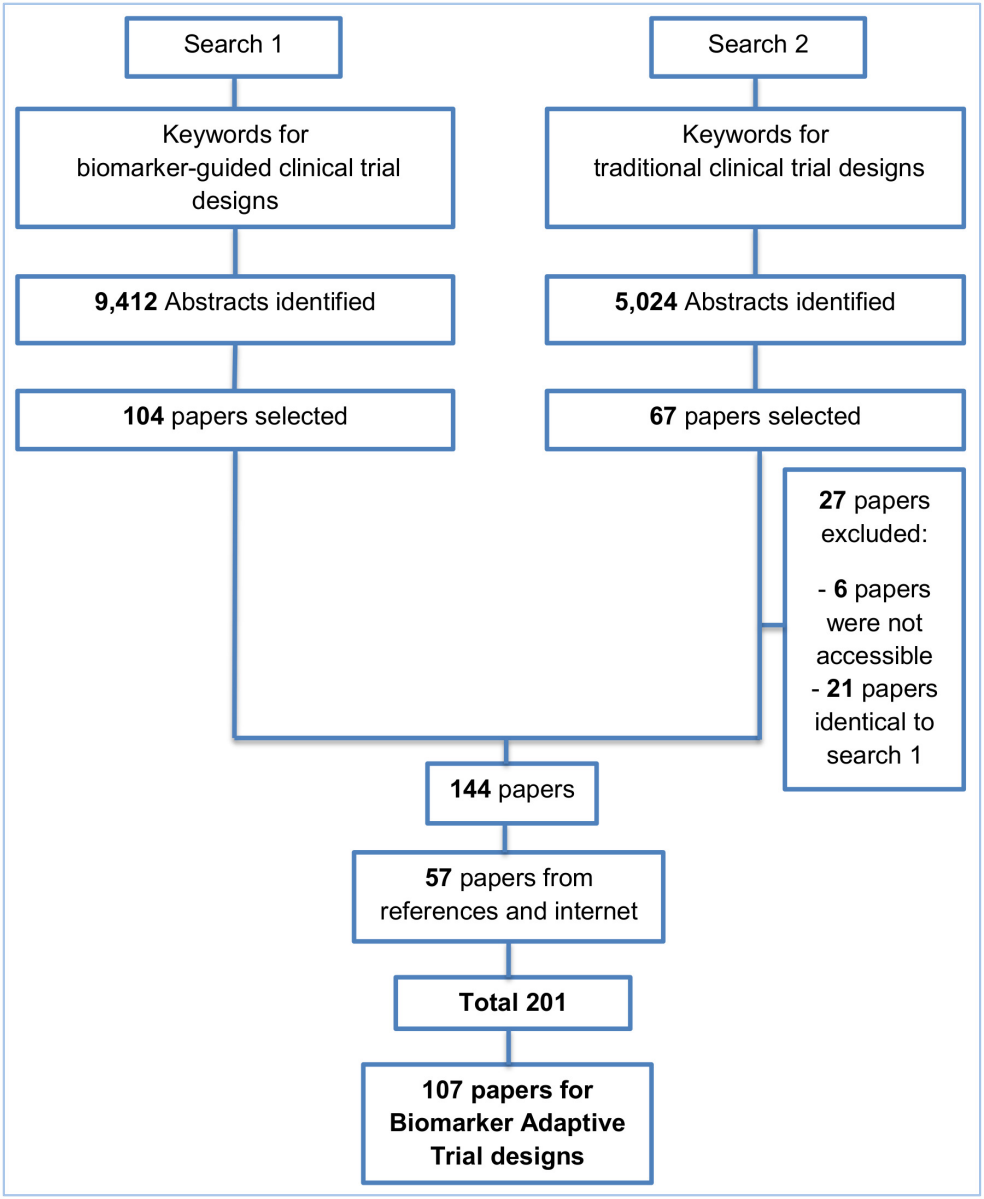
CONSORT diagram of the review process.

We have undertaken a search of the MEDLINE (Ovid) database, restricted to published papers in the English language within the previous ten years aiming to identify articles which describe and discuss both non biomarker-guided trial designs, which we will refer to as ‘traditional’ trial designs, and biomarker-guided trial designs. Two separate strategies as illustrated in Fig 1 were used to identify relevant articles, and the keywords utilized in the search are presented in S1 Keywords. First author (MA) screened the available titles and abstracts, and second and third authors (ALJ, RKD) were consulted where it was questionable whether a paper should be included or not. Our initial search resulted in 9,412 and 5,024 relevant titles for biomarker-guided clinical trial designs and traditional designs respectively. From the 9,412 papers, 104 articles were included based on their title and abstract. From the 5,024 papers, 40 articles were included based on their title and abstract and after removing inaccessible articles or those already identified in the search for biomarker-guided trial designs. A further 57 papers were identified from searching both the reference list of included articles and the internet (the internet searches were performed using the same keywords as those for the Ovid strategy). Of the 201 included papers, biomarker-guided adaptive trial designs were referred to in 107 papers. An extraction form was designed to collect all necessary information, and the summary of the extracted data was reviewed by the second and third authors (ALJ, RKD). If there were any ambiguities or confusion as to the extracted data, the second and third authors were consulted. For each included paper, the following details were extracted: definition of the trial design(s) referred to in the paper, how patients were screened and/or randomized based on their biomarker status, treatment groups randomized to, other key information relating to the trial design and methodology, advantages and limitations, and examples of actual trials which have adopted designs if mentioned together with the proposed methodology and clinical field where the design had been applied. There is no evidence of some of proposed trial designs in practice in the literature which was used for our review; however, they may well currently be in use in ongoing trials. The review of all trial designs which have been implemented in practice is beyond the scope of this paper.

### Adaptive designs

Before discussing the specific biomarker-guided adaptive trial designs, we consider key aspects of adaptive trial designs in more general.

#### Definitions and terminology

To date, several authors have given different definitions about adaptive designs in general [34–36]. Chow et al. (2005) [34] described the adaptive design as a strategy that allows adaptations in trial procedures and/or statistical procedures after initiation of the trial without undermining the validity and integrity of the trial. In 2006, the Pharmaceutical Research Manufacturer Association (PhRMA) Working Group on Adaptive Design [35] defined an adaptive design as a clinical trial design that uses accumulating data to decide how to modify aspects of the study as it continues, without undermining the validity and integrity of the trial.

In 2010, US Food and Drug Administration defined an adaptive design as a study that includes a prospectively planned opportunity for modification of one or more specified aspects of the study design and hypotheses based on analysis of (usually interim) data from subjects in the study [36]. In the context of biomarker-guided therapies, Chen et al. (2014) [12] defines the biomarker adaptive trial design as “designs which identify most suitable target subpopulations with respect to a particular treatment, based on either clinical observations or known biomarkers, and evaluate the effectiveness of the treatment on that subpopulation in a statistically valid manner”.

Some researchers refer to these approaches as flexible designs [33, 37–40], terminology which can cause confusion since some trial designs which allow adaptivity are by no means flexible, for example those with pre-specified rules in terms of how to proceed based on interim data analyses [41]. Thus, the term ‘flexible designs’ can include designs with both planned and unplanned properties [42].

#### Adaptations to the design

Adaptations based on interim analysis, which are made during the course of an adaptive strategy include adding or dropping treatment arms, changes in the required sample size, changes in the allocated proportion of the study population in order to randomize more patients to treatment arms which are doing better, or refinement of the existing study population according to their predictive biomarkers [38–40, 43–49].

In Personalized Medicine the most common adaptations during the implementation of adaptive designs refer to changes in randomization probabilities within the biomarker-defined subgroups or dropping a biomarker-defined subgroup [15, 50].

Generally, this type of biomarker-guided approach is appropriate when (i) the candidate biomarker is not known at the start of the study; (ii) there are multiple experimental treatments and pre-specified biomarker-defined subgroups; (iii) existence of well-established analytical validity; (iv) rapid turnaround time for biomarker assessment [12, 15, 51].

#### Analysis of adaptive designs

Although both a Bayesian and Frequentist framework has been used for the analysis of adaptive designs [26, 52–54] the former has been described by many authors as a more suitable perspective due to its flexibility as it enables revision of knowledge according to prior information. I-SPY2 and BATTLE studies are examples of actual adaptive trials designed with a Bayesian framework [48, 49, 55]. Nevertheless, the Bayesian perspective in adaptive designs can cause many problems in terms of computational demands, inference making and parameter estimations [10, 26, 55, 56].

### Biomarker-guided adaptive trial designs

**Fig 2:**
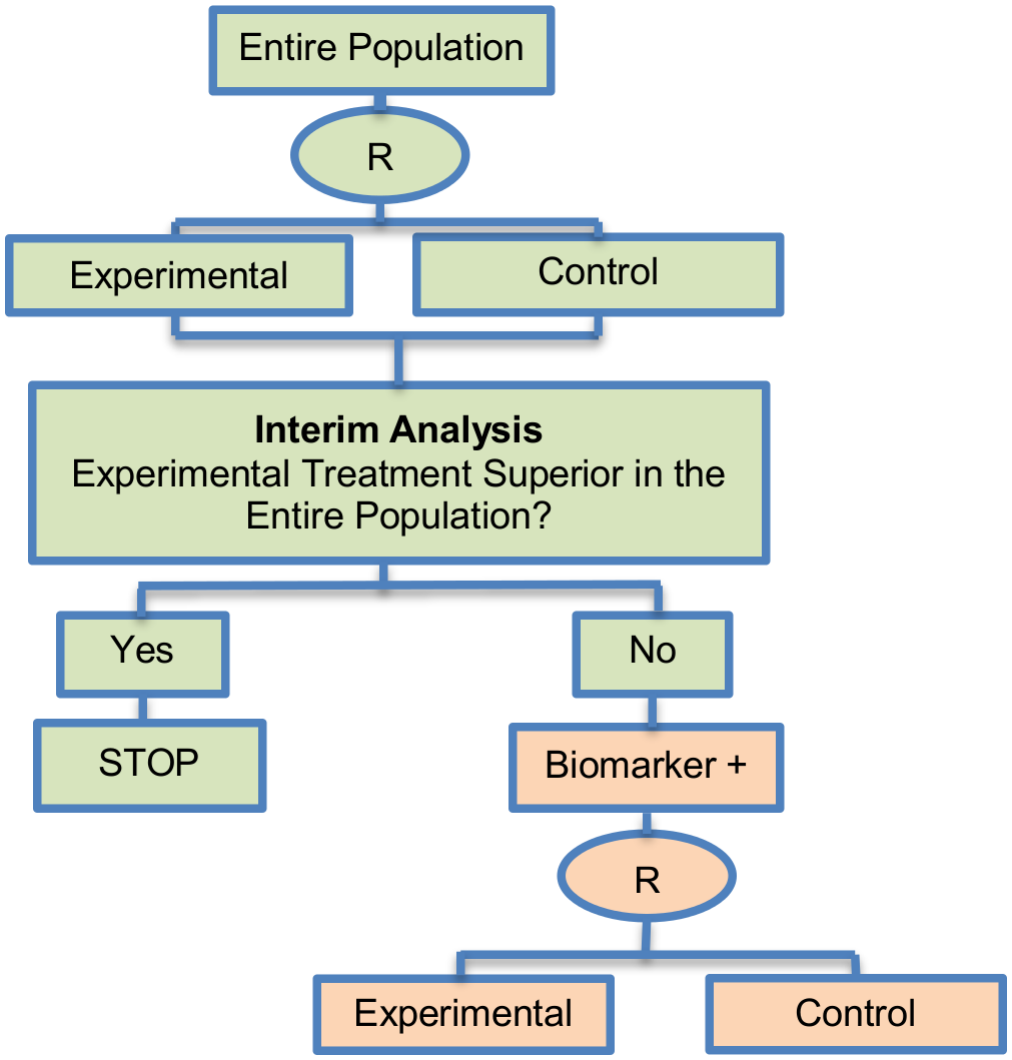
Adaptive signature design. “R” refers to randomization of patients.

**Fig 3:**
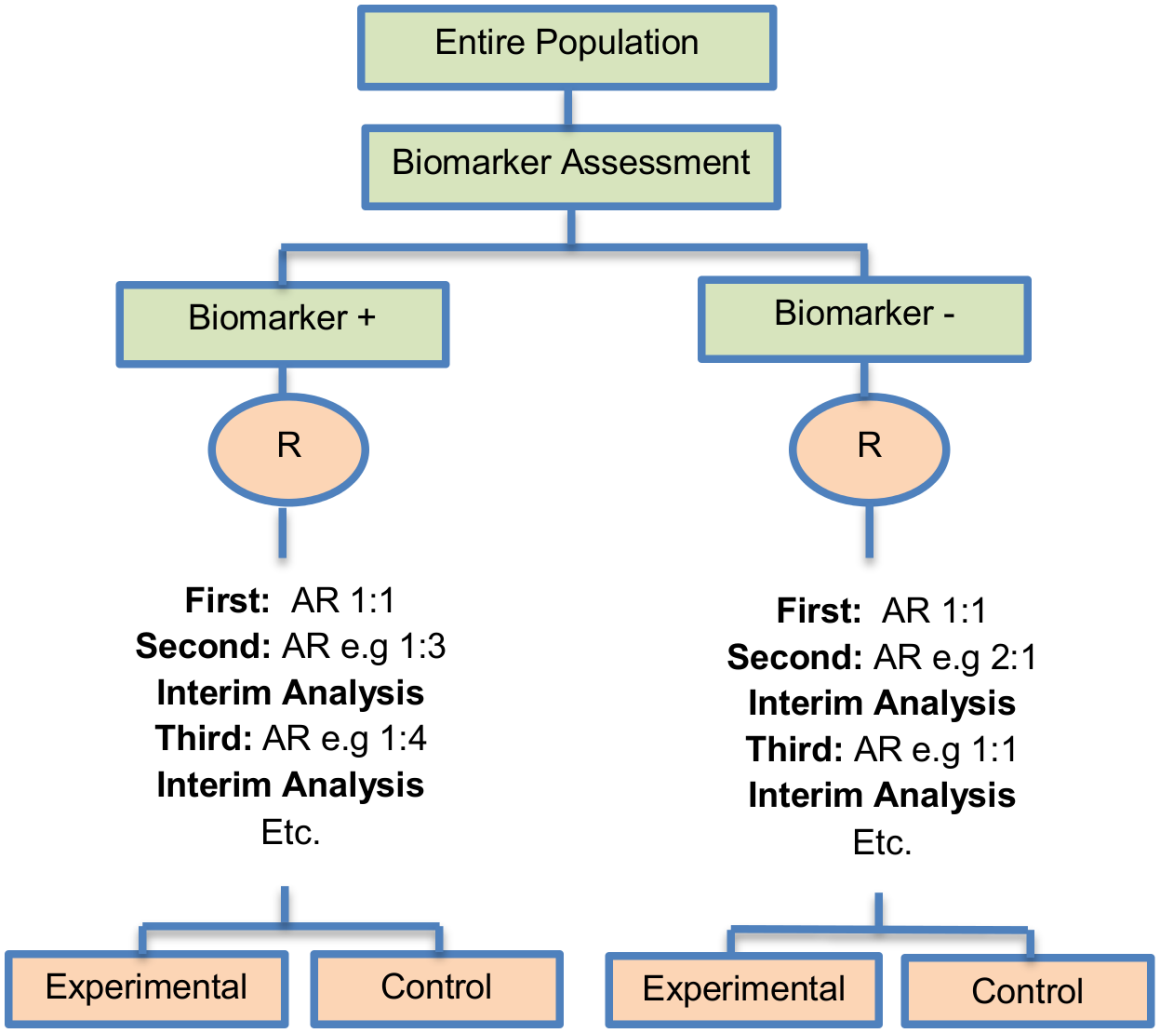
Outcome-based adaptive randomization design. “R” refers to randomization of patients.

**Fig 4:**
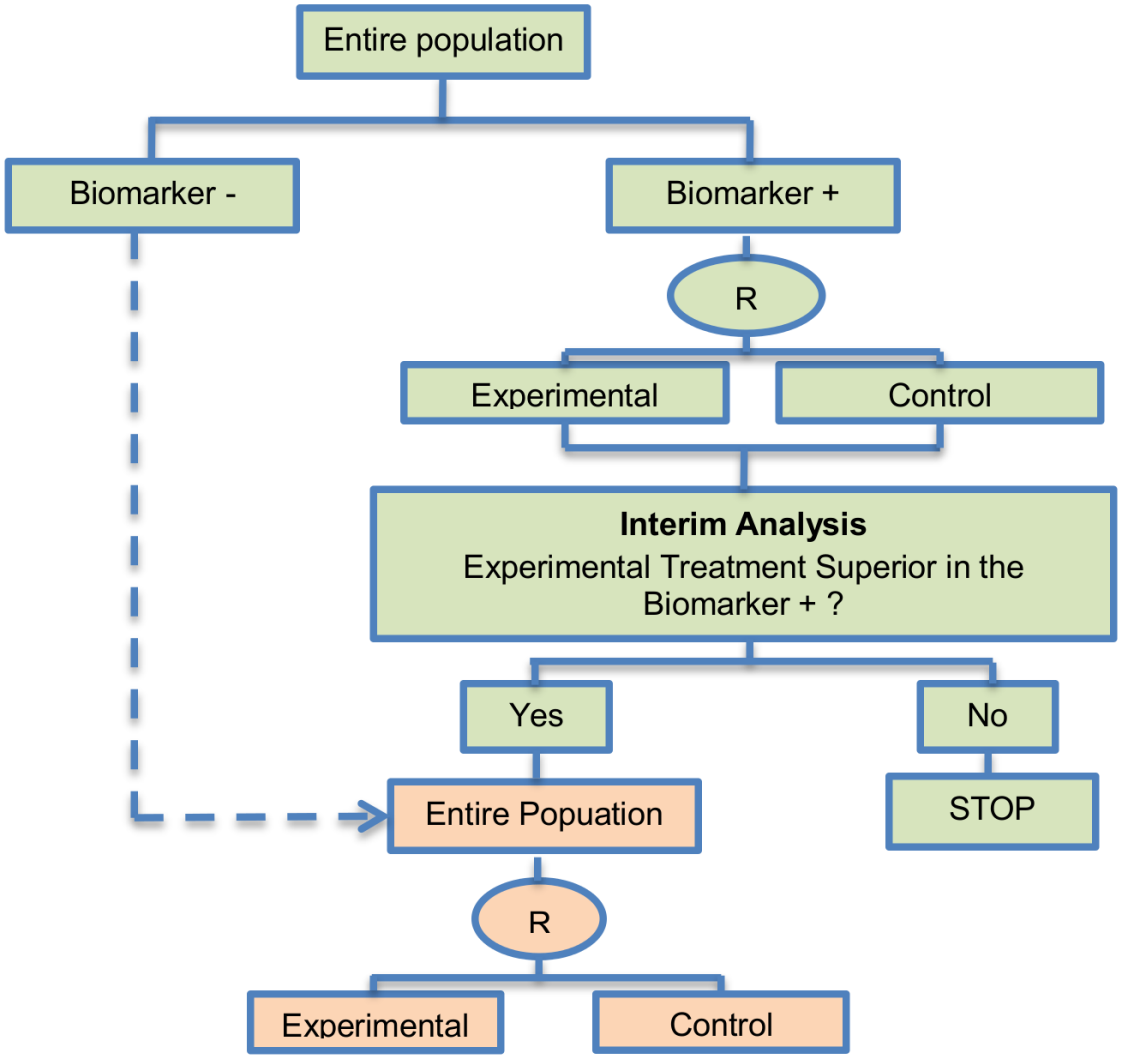
Adaptive threshold sample-enrichment design. “R” refers to randomization of patients.

**Fig 5:**
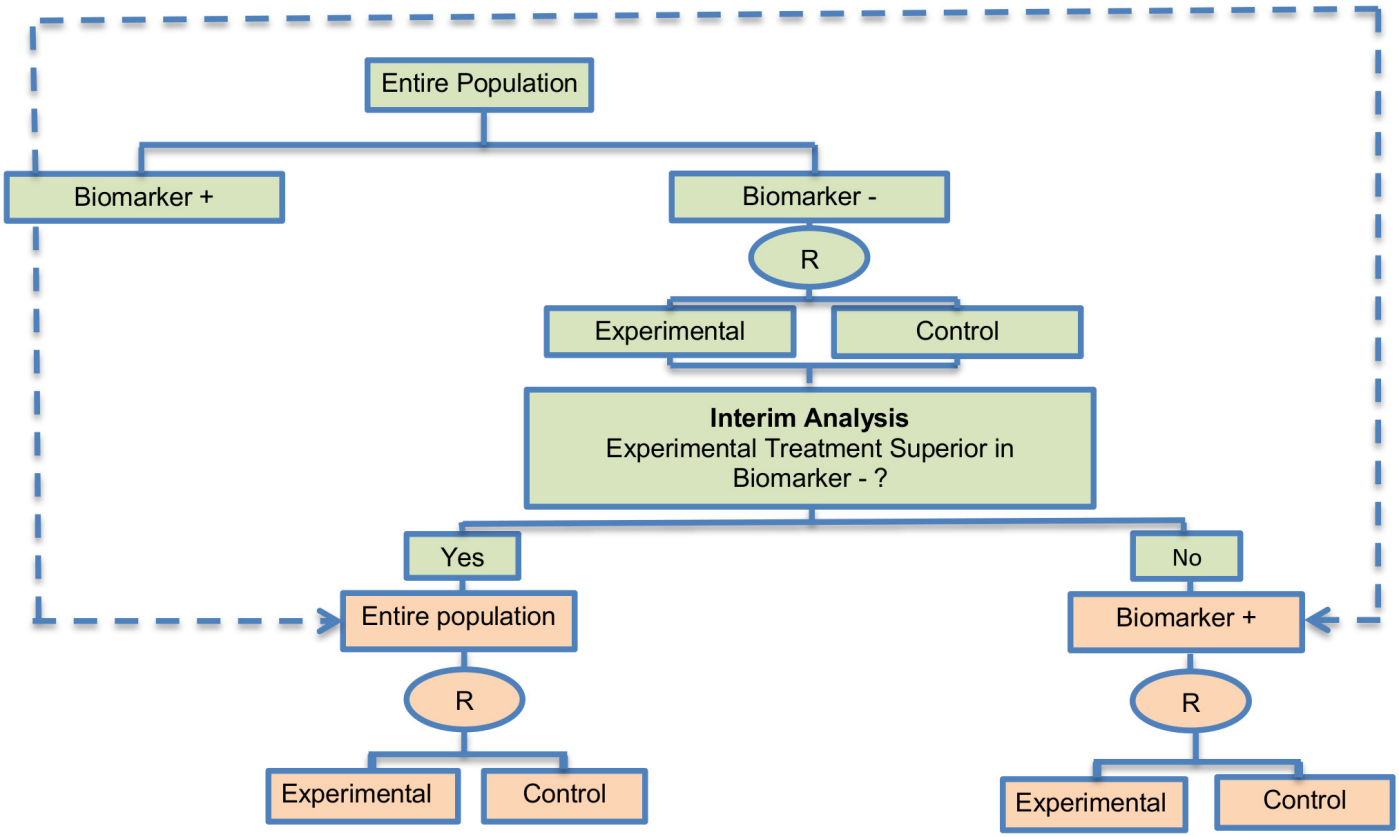
Adaptive patient enrichment design. “R” refers to randomization of patients.

**Fig 6:**
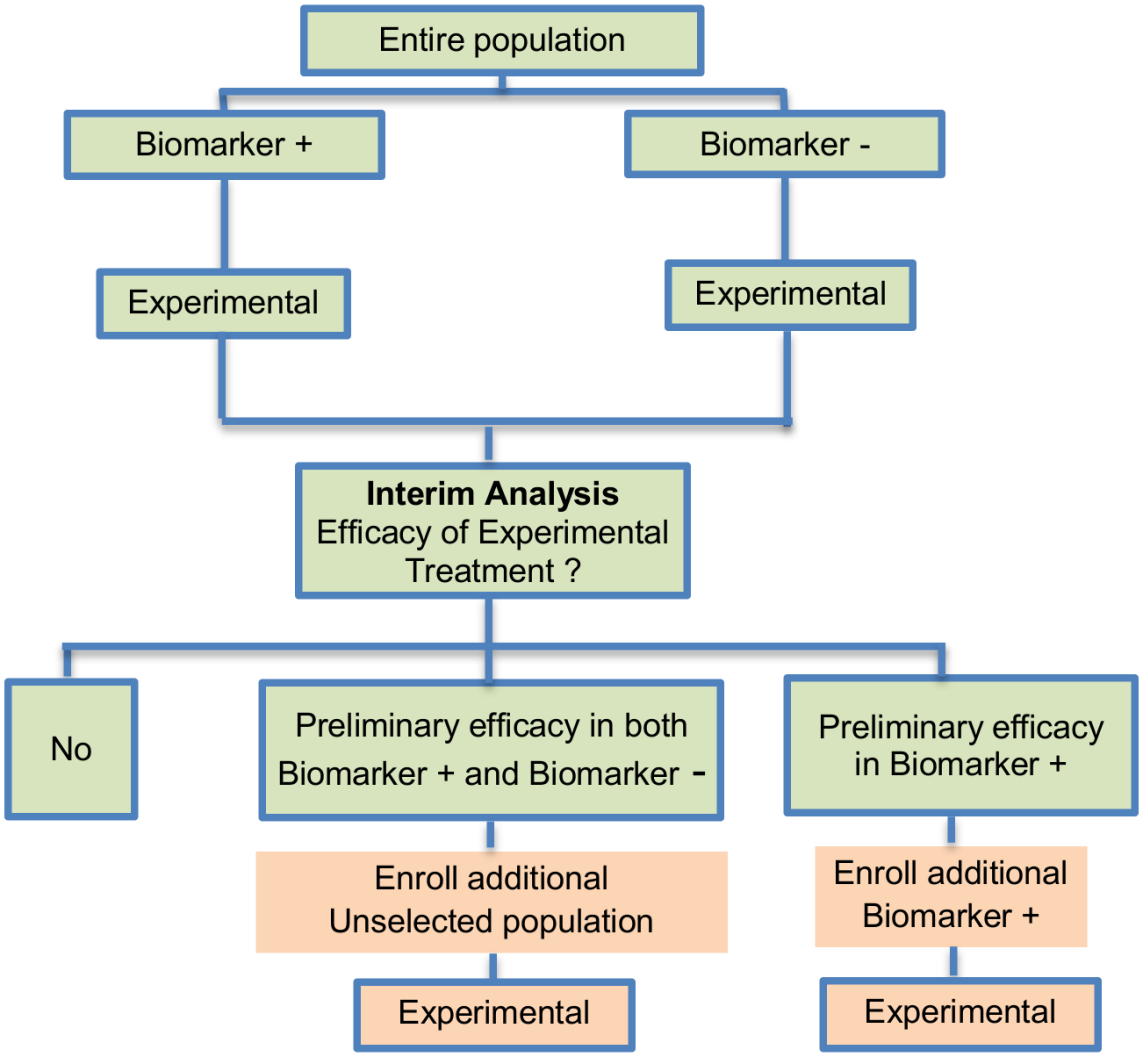
Adaptive parallel Simon two-stage design. “R” refers to randomization of patients.

**Fig 7:**
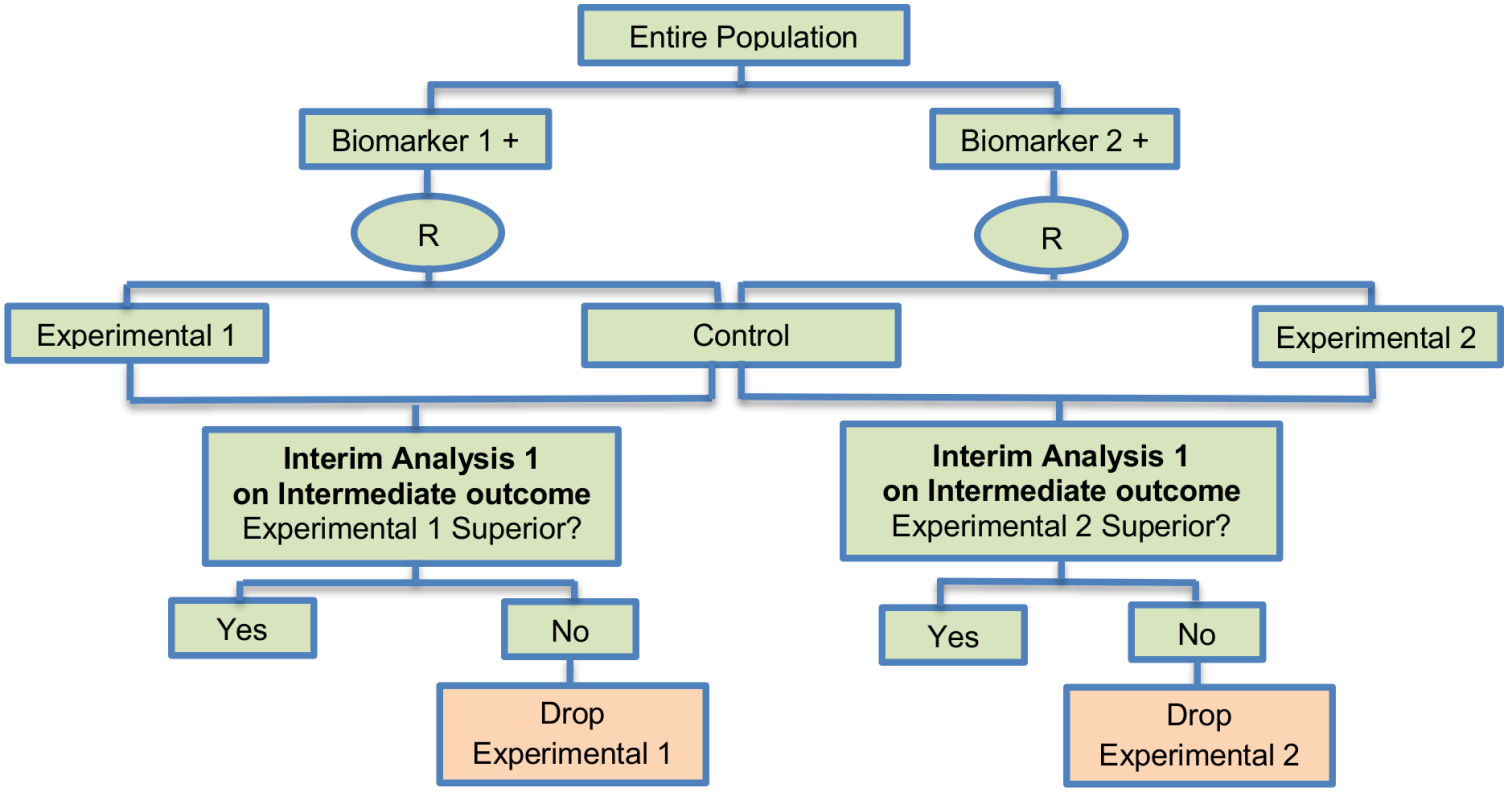
Multi-arm multi-stage (MAMS) design. “R” refers to randomization of patients.

**Fig 8:**
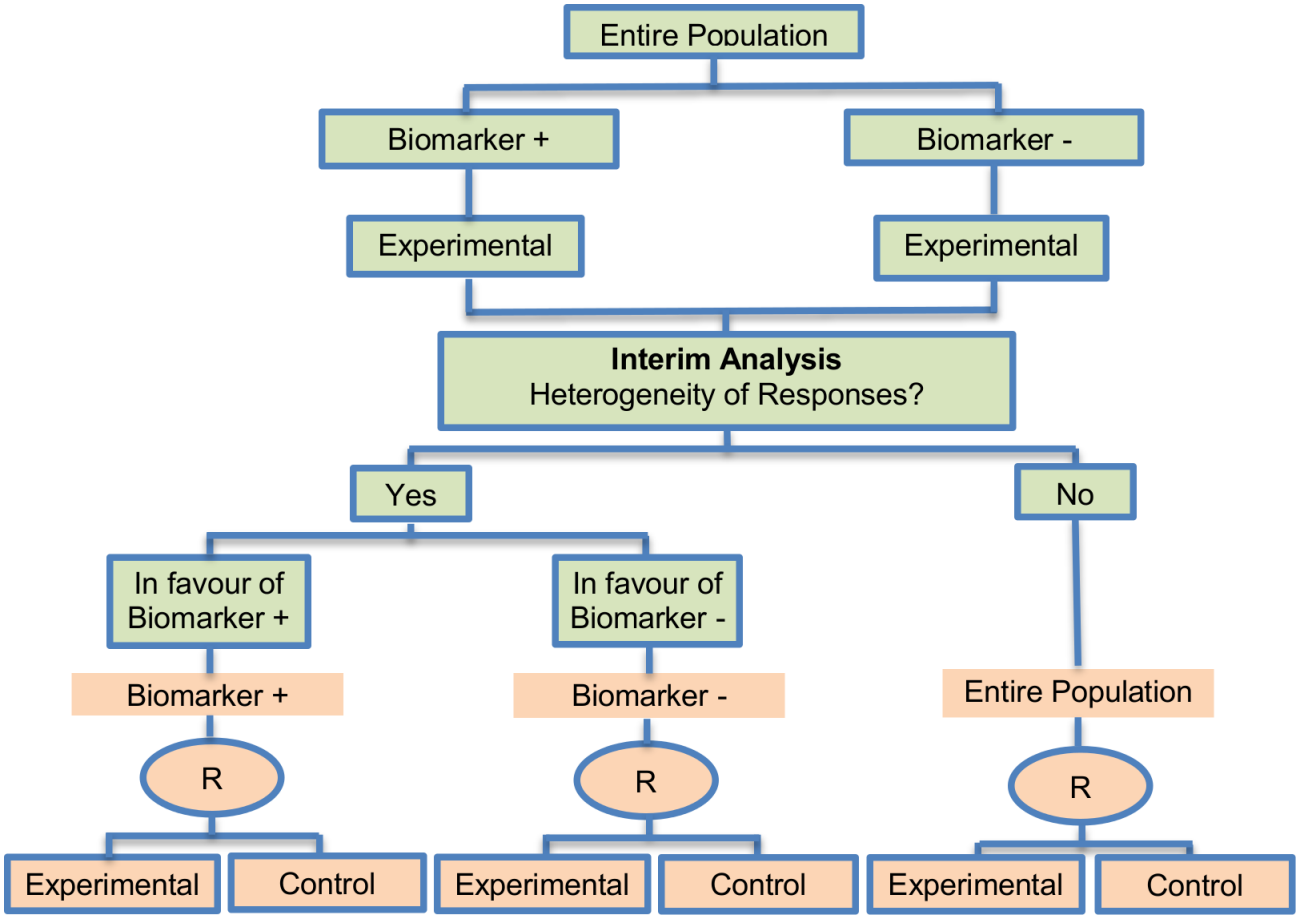
Stratified adaptive design. “R” refers to randomization of patients.

**Fig 9:**
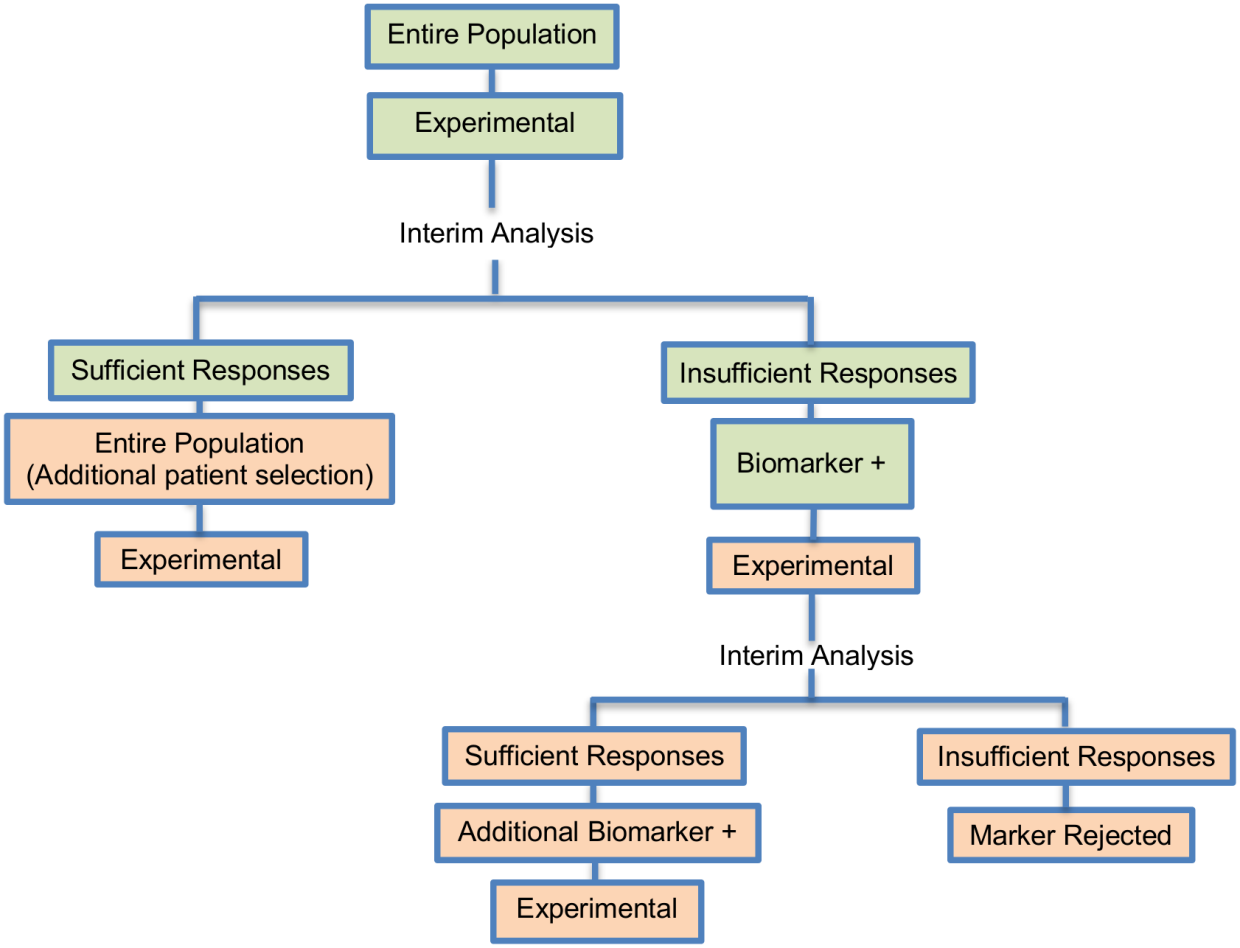
Tandem two stage design. “R” refers to randomization of patients.

In our review, we have identified eight main biomarker-guided adaptive designs. Four of the eight designs also have variations. Each main design is presented graphically in Figs 2–9. The characteristics and methodology of the eight main designs are discussed below and summarized in Table 1, whilst information on their variations are summarized in S1 File (Table A in S1 File).

**Table 1:** Characteristics of biomarker-guided adaptive trial designs in Phase II and Phase III.

Types of Biomarker-guided adaptive trial designs	Phase	Adaptations	Pros	Cons
**Adaptive signature design** **(30 papers)** [2, 6, 8, 9, 12, 14–16, 18, 20, 21, 24, 27, 31, 32, 47, 49, 57, 58–68]	III	Identification of biomarker-positive subpopulation	Identification of optimal group of patients which benefit the most from a specific treatment.	Larger sample size may be required, especially when there is small difference between biomarker-negative and biomarker-positive patients.
**Also called**: Two-stage Adaptive Signature design, Adaptive Two-stage design, Biomarker-Adaptive Signature design			Identification and validation of candidate biomarker in a single trial.	Can limit its power when testing the treatment effectiveness in the biomarker-positive subgroup as half of patients are used for signature development and half for validation of the biomarker.
			Avoids inflation of type I error rate as it does not use the individuals on which the predictive signature was developed in order to test the treatment effect.	Treatment comparisons can only performed when the study is completed.
			Rapid and efficient approval of the novel treatment.	
			No modifications in randomization weights or in eligibility criteria are made, consequently, it avoids any statistical adjustment needed to ensure that there is no introduction of bias.	
**Outcome-based adaptive randomization design** **(24 papers)** [14, 26, 29, 32, 37, 40, 47, 49, 52, 56, 59, 62, 63, 65, 69, 70–78]	II	Change in randomization ratio	Smart, novel, and ethical approach	Complexity in terms of building-up the trial design, conduct and analysis of the trial.
**Also called**: Adaptive randomization Bayesian Adaptive, Bayesian Adaptive randomization, Combined dynamic multi-arm, Outcome-Adaptive randomization, Outcome-based Bayesian Adaptive Randomization			Permits updating patient’s outcome (it uses the accumulated information in order to assign patients to different treatment arms; the arm which seems to benefit the study population the most, is composed of the higher proportion of randomized individuals).	Can make incorrect decisions in case of incorrect biomarker selection as the design is based on the accumulated data about how well the biomarker performs.
			Can result in high probability of success of the trial as there is increase in the number of patients who receive effective treatments.	Requirement of relatively short biomarker and endpoint assessment (quick testing of the biomarker is required in order to avoid incorrect decision regarding the assignment of patients and rapid assessment of outcome to randomize adaptively according to the updated outcome.).
			In the Bayesian perspective, Type I and II errors can be controlled by carefully calibrating the design parameters.	Likely to introduce bias due to time trends in the prognostic mix of individuals enrolled to the study (e.g., less frail individuals considered for the trial after some point due to toxicity concerns).
			Can boost patients’ ethics as patients are assigned to the best available therapy.	
**Adaptive threshold sample-enrichment design** **(5 papers)** [18, 20, 21, 63, 79]	III	Change in the inclusion criteria of the study population after the initial stage of the study in order to broaden the targeted patient population.	More cost-effective as it avoids further recruitment of patients when there is no difference in treatment outcome among the biomarker-defined subgroups.	Cannot work if there is no information about a subset of patients for whom the novel treatment is more effective than others before the beginning of the trial.
**Also called**: Threshold sample-enrichment approach, Two-stage Sample Enrichment, Two-stage sample-enrichment design strategy			Researchers can use the data which was accumulated during the first stage of the study to proceed with further investigation of any other potential assumption made at the start of the trial.	
**Adaptive patient enrichment design** **(23 papers)** [3, 6, 7, 14, 18, 20, 21, 29, 38, 42, 43, 63, 70, 74, 78, 80–87]	III	Information obtained from interim stage is used to broaden the targeted patient population.	Can detect a particular biomarker-defined subgroup most likely to respond to the novel treatment, thus the efficiency of study design can be increased.	Can be quite complex.
**Also called**: Adaptive accrual, Adaptive accrual based on interim analysis design, Adaptive Enrichment, Adaptive Modification of Target Population. Adaptive Population Enrichment, Two-stage Adaptive Design, Two-stage adaptive accrual			Can gain more power than a fixed study design under the scenario that the genomic biomarker is predictive of treatment effect (i.e., the value of effect size indicates that there is treatment effect in the biomarker-defined subgroup, e.g. the value of 0.4) than in the case where the genomic biomarker is prognostic (i.e., the scenario where we assume that the value of effect size is zero).	Can result in biased treatment effect estimates.
				Criticised as a design without satisfactory operating characteristics in real practice with a lack of generalizability and information in subgroups which are excluded.
				May augment the duration of the trial depending on the prevalence of the biomarker for the biomarker—defined subgroup which continues to full accrual due to recruitment of many more biomarker-positive patients.
				Requirement of an appropriate futility boundary and rapid and reliable clinical endpoint.
				Conservativeness of futility boundaries as the futility boundary is set to be in the region in which the observed efficacy of the standard of care is greater than that for the experimental treatment.
				Assumes complete confidence in the biomarker.
				Early termination of the entire trial is not permitted.
**Adaptive parallel Simon two-stage design** **(8 papers)** [6, 76, 85, 88–92]	II	The design starts with two parallel studies and according to the results of the initial stage we enroll selected or unselected patients during the second stage.	May reduce the required sample size.	Does not allow early termination of the trial for efficacy in biomarker-defined subgroups during the first stage of the trial.
**Also called**: Biomarker-adaptive parallel two-stage, Adaptive parallel, Two-parallel Simon, Two-stage design			May augment the efficiency of the trial as it allows for early understanding that a particular experimental treatment is beneficial in a specific biomarker-defined subgroup.	
			Straightforward and simple strategy with reasonable operating characteristics.	
**Multi-arm multi-stage designs** **(16 papers)** [18, 20, 40, 56, 63, 69, 89, 93–103]	II/III	Experimental arms can be dropped for futility from the study.	Promising treatments are tested concurrently using a smaller number of patients as some treatments arms can be dropped early for futility.	High setting-up time due to the complexity caused by logistic issues and collection of experimental drugs from different companies.
**Also called**: Adaptive biomarker-driven design, Adaptive Analysis, Adaptive Multi-stage designs, Multi-stage			Reduced costs and time as they assess multiple treatments simultaneously.	Operational challenges regarding the randomization and the modifications of allocation ratios after the performance of an interim analysis.
			Preferable to continue with the investigation of promising treatments as compared to the conduct of separate single-arm phase II clinical trials.	
			The simultaneous assessment of multiple experimental treatments increases the chance of identifying a promising treatment.	
			It is unlikely that the trial will stop for futility as multiple experimental treatments are tested and hence, it is not likely that all experimental arms will be ineffective and dropped.	
			Can ease the regulatory and administrative burden as compared to building—up separate trials.	
			Unpromising experimental arms can be dropped in a quick and reliable way.	
**Stratified adaptive design** **(1 paper)** [89]	II	The number of patients and decision rules are based on the observed response rates during the first stage of the study.	Can avoid unethical studies in patients for whom the novel treatment is not effective as it allows for the identification of efficacy which is limited to a particular biomarker-defined subgroup.	No information found
No alternative names found for this trial design			The trial can continue to Phase III only with a subgroup which is proven to benefit from the experimental therapy and not with the entire population.	
			Less numbers of individuals for whom the novel treatment is not effective will be tailored to toxic treatments.	
			Permits the identification of the actual treatment benefit in at least one biomarker-defined subgroup.	
			Avoids the termination of tailoring a novel treatment due to treatment effect dilution in the entire population.	
			Permits early stopping of efficacy or inefficacy.	
**Tandem two stage design** **(5 papers)** [21, 63, 76, 90, 104]	II	Assessment of treatment effectiveness in the entire population at the first stage of the study to make a decision about enriching the targeted patient population.	Although the two stages could be run separately, i.e. one for the biomarker-positive subgroup and the other for the unselected patients, the performance of the study in this way can increase the duration and costs of the trial. Consequently, it will be better to run the study in just one trial so as to have a more seamless study.	No information found
**Also called**: Tandem two-step phase II trial, Tandem-two step trial (phase II), Tandem two-step phase 2 trial design, Tandem two-step			Allow estimating response rates not only in the unselected biomarker-defined patients (entire population) but also in the biomarker-positive subset.	
			Identify whether the experimental treatment is beneficial in the entire population, and if it is not, then can test whether the candidate predictor can enrich the responding population.	
			Allow for simultaneous testing of multiple different biomarkers for the same treatment in a single parallel multi-arm trial.	

#### Adaptive signature design

The adaptive signature design is described in 30 (28%) papers of our review. It is a two-stage Phase III non-Bayesian trial design proposed by Freidlin and Simon (2005) [105] for settings where an assay or signature that identifies sensitive patients (i.e, biomarker-positive patients) is not known at the outset. This trial design permits the development and evaluation of a biomarker based on high dimensional data. It uses a training set to identify predictive biomarkers and evaluates them in a validation set. Generally, this approach is useful when there is no available biomarker at the start of the trial or there is a great number of candidate biomarkers which could be combined to identify a biomarker-defined subgroup, and the attention is given first to the entire study population. Five variations of the adaptive signature design have also been identified, with differences occurring mainly in terms of the analysis. These variations are the following: i) Adaptive threshold design, ii) Molecular signature design, iii) Cross-validated Adaptive Signature design, iv) Generalized adaptive signature design and v) Adaptive signature design with subgroup plots. Information about each variant can be found in S1 File, section ‘‘Variations of Adaptive signature design”.

**Design**: Fig 2 graphically represents the trial design. The design begins with a comparison between the experimental treatment and the standard treatment in the entire study population at a pre-specified level of significance. In case that the overall result is positive, it is considered that the treatment is beneficial and the trial is closed. If the comparison in the overall population is not promising, then the entire population is divided in order to develop and validate a biomarker, using a split sample strategy. More precisely, a portion of patients is used to detect a biomarker signature that best distinguishes subjects for which the novel treatment is better than the standard treatment. Hence, this approach (i) identifies patients who are more susceptible to a specific treatment during the initial stage of the study (at the interim analysis); (ii) it assesses the global treatment effect of the entire randomized study population through a powered test, and (iii) finally, it assesses the treatment effect for the biomarker-positive subgroup identified during the initial stages of the study but only with patients randomized in the remainder of the trial, the so-called ‘validation test’.

**Methodology**: The analysis is undertaken as follows: At the interim analysis stage, if the overall treatment effect is not significant at a reduced level *a*_1_ (< 0.05), the full set of P patients in the clinical trial is partitioned into a training set Tr and a validation set V. A pre-specified algorithmic analysis plan is applied to the training set to generate a classifier Cl(x;Tr) where x is a biomarker vector. This classifier is a function that identifies a biomarker-positive subgroup of patients who appear to benefit from the experimental treatment E. Cl(x;Tr) = 1 means that a patient with x is predicted to benefit from E whereas Cl(x;Tr) = 0 indicates that a patient is not predicted to benefit from E [57]. At the final analysis, the experimental treatment E is compared with the standard of care (or control) treatment in the biomarker-positive patients subgroup using a significance level of *a*_2_ = *α*−*a*_1_ in order to ensure that the overall likelihood to obtain a false-positive conclusion is no greater than *α* (= 0.05).

Freidlin and Simon (2005) [105] recommended that a level of *a*_1_ = 0.04 (two-sided) is allocated to the entire population hypothesis and *a*_2_ = 0.01 (two-sided) is allocated to the biomarker-positive subgroup hypothesis. The multiplicity problem is a concern with this approach as the statistical test is conducted twice. The power of this strategy can be increased using K-fold cross-validation as Freidlin and Simon (2005) [105] demonstrated (see the Cross-validated adaptive signature design (CVASD) in S1 File for further information).

**Statistical/practical considerations**: Although the adaptive signature design allows for approval of the novel treatment in a quick and efficient way, the main statistical challenges to be taken into account include the potential increase in the number of patients and the limited power to assess the treatment effect in the biomarker-defined subgroup. However, this approach avoids introduction of bias since the adaptations do not involve modifications in allocation ratio and eligibility criteria. Further, it prevents the inflation Type I error rate as the design does not use the study population which was employed to develop the predictive signature for the assessment of the treatment effect.

#### Outcome-based adaptive randomization design

The outcome-based adaptive randomization approach is referred to in 24 (22.4%) papers. In the context of personalized medicine, this design is used when the biomarkers are only putative or not known at the beginning of a Phase II trial and is also useful when there are multiple targeted treatments and biomarkers to be considered. It aims to test simultaneously both biomarkers and treatments while providing more patients with effective therapies according to their biomarker profiles. Outcome-adaptive randomization is sometimes included under the umbrella of “Bayesian clinical trials” but as criticized by Korn and Freidlin (2011) [71], there is nothing inherently Bayesian about it. There is a single variant of the outcome-based Adaptive Randomization design with differences occurring in its analysis methodology. This variant is referred to as Bayesian covariate adjusted response-adaptive randomization and information about this approach can be found in S1 File, section ‘‘Variation of Outcome-based adaptive randomization design”. Two examples of actual trials which use the outcome-based adaptive randomization approach are the following: i) BATTLE trial [14, 29, 52, 59, 62, 70, 72–74, 76, 77], ii) ISPY2 [29, 32, 49, 62, 72, 75, 76].

**Design**: An illustration of this approach is shown in Fig 3. The trial begins with the assessment of patients’ biomarker status. The design permits the modification of patients’ allocation to different treatment arms so that the arm(s) which seem(s) to benefit the study population the most, is composed of the higher proportion of randomized patients. Consequently, we have randomization probabilities which do not stay fixed over time (e.g. change from adaptive randomization (AR) ratio 1:1 to AR 2:1, see Fig 3). The random assignment of patients to treatment arms, according to their biomarker status, depends on the use of accumulated patients’ data about how well the biomarker performs as at each interim analysis stage. When these accruing outcome data indicate that an experimental treatment is more effective as compared to the standard of care (or control), it is possible that a higher number of patients will be assigned to this particular experimental arm.

**Methodology**: Zhou *et al*. (2008) [77] proposed an analysis plan in a Bayesian hierarchical framework using the Bayesian probit model to characterize the disease control rate for each treatment by biomarker subgroup. Therefore, the estimates for the treatment and the biomarker effect are provided by using the adaptive randomization design with the incorporation of a hierarchical Bayes model (it is a probit model included in the category of generalized linear models which uses the probit link function to model categorical or ordinal data). More precisely, the process starts with the biomarker profile assessment of all eligible patients and then according to the profile of each individual, the study population will be assigned to the different biomarker groups (e.g. a patient with a particular biomarker will be assigned to a specific biomarker group). Due to the fact that at the beginning of the trial we do not know the true disease control rate (i.e., the proportion of patients who demonstrate response to a treatment) the trial begins with equal randomization so that each treatment by biomarker subgroup is composed of at least one individual with a known disease control status (whether the patient will experience progression given a certain treatment). Next, the trial continues with adaptive randomization of patients; this is achieved by using the Bayesian probit model to calculate the posterior disease control rate. After the posterior rate is found, we define the randomization rate as the posterior mean of the disease control rate of each treatment in each biomarker-defined subgroup. The adaptive randomization process continuous until the last individual is enrolled and can stop early only in case that all treatments are dropped due to inefficacy. Whereas in many trial designs the baseline covariate (in this case the biomarker) is considered as prognostic, the design proposed by Zhou et al. (2008) [77] allows for modelling the treatment by biomarker interactions where the biomarker is in fact predictive. The incorporation of the above hierarchical Bayesian structure allows ‘borrowing strength’ or information-sharing across patients receiving the same treatment but with different biomarker profiles [77].

**Statistical/practical considerations**: Despite the fact that this design can be considered successful as an ethical approach where patients can be assigned to the most effective treatments according to their biomarker profiles, an issue that raises concern is the requirement of a relatively short assessment period of both biomarker and endpoint to avoid erroneously not only the assignment of patients but also the adjustment of the randomization rate. Also, potential introduction of bias due to time trends in the prognostic mix of the patients enrolled to the study should be taken into consideration.

#### Adaptive threshold sample-enrichment design

Adaptive threshold enrichment design was identified in 5 papers (4.7%) of our review. This approach is a two-stage design in a Phase III setting which was proposed by Liu et al. (2010) [79] to adaptively modify accrual in order to broaden the targeted patient population (see Fig 4).

**Design**: The design is based on the former knowledge that a specific biomarker-defined subgroup (biomarker positive) is believed to benefit more from a novel treatment as compared to the remainder of the study population (biomarker negative). This knowledge can be gained, for example, from previous studies such as a large scale comparative trial (Phase III) when there was treatment effect heterogeneity among the study population. This design allows the trial to be terminated for futility in the biomarker positive subgroup. More precisely, the trial proceeds as follows: (i) accrue and randomize only biomarker positive patients; (ii) conduct an interim analysis in order to compare the experimental treatment with the standard of care within the biomarker positive subgroup; (iii) if the interim result is negative, then the accrual stops and the trial is closed without showing a treatment benefit; if the result is ‘promising’ for the specific biomarker-positive subgroup, then the study continues with this specific biomarker positive subgroup and accrual also begins for biomarker negative patients. Thus, the trial continues with patients randomized from the entire population. A ‘promising’ result in the biomarker positive subgroup at the interim stage is claimed when the estimated treatment effect is above a particular pre-specified threshold.

**Methodology**: The analysis is undertaken as follows: At the interim analysis stage, the treatment effect of a sample of patients (*n*_1_) from the biomarker-positive subset is estimated. If an improvement is seen in the experimental treatment arm which is greater than a pre-specified threshold value (i.e. the estimated treatment difference between the novel treatment arm and the control treatment arm for this subpopulation is greater than a threshold value *c* divided by the square root of the aforementioned sample size *n*_1_) the trial continues with accrual of patients from the entire biomarker-positive subgroup and additional patients are also accrued from the biomarker-negative subpopulation; otherwise the trial is stopped for futility. At the end of the trial, the treatment effect is estimated for all subpopulations. Researchers should choose the sample size *n*_1_ so that a persuasive result can be reached when the first stage of the trial is completed. In general, the threshold value *c* can be determined so that $c/\sqrt{n_{1}}$ is a proportion of the smallest meaningful treatment improvement that researchers expect, e.g. it can be set to half of the smallest clinically important difference. Other methods also have been proposed [79].

Liu et al. (2010) [79] give a detailed description regarding the statistical formalization of the Type I error rate of this two-stage test and the power for assessing group-specific treatment effects. Also, Liu et al. (2010) [79] give detailed information on testing hypotheses based on the overall treatment effect indexed as a weighted average of the group-specific treatment effects, where the weight can be specified as the prevalence of that particular subgroup.

**Statistical/practical considerations**: The Adaptive threshold sample-enrichment design is not feasible if there is no prior knowledge regarding a subgroup of patients which is more susceptible to a particular treatment than others. In addition, this approach is considered more cost-effective as there will be no further recruitment from the study population when there is no evidence of treatment effectiveness.

#### Adaptive patient enrichment design

The adaptive patient enrichment design was included in 23 papers (21.5%). This is a two-stage Phase III clinical trial design proposed by Wang et al. (2007) [80]. There is a single variant of the adaptive patient enrichment design with differences occurring in its methodology. This variant is referred to as Modified Bayesian version of the two-stage design of Wang et al. (2007) [80] and information about it can be found in S1 File, section ‘‘Variation of Adaptive patient enrichment design”. An example of actual trial which incorporates the adaptive patient enrichment design is the NCT01001234 trial [42, 87].

**Design**: This approach is used for the comparison of an experimental treatment with the standard of care (control) which adaptively modifies accrual to two predefined biomarker-defined subgroups based on an interim analysis for futility. Fig 5 presents the adaptive patient enrichment design, and in general it flows as follows: (i) accrue both biomarker-positive and biomarker-negative patients; (ii) perform an interim analysis to evaluate the experimental treatment in the biomarker-negative subgroup; (iii) if the interim result in that subgroup is ‘not promising’, defined as the observed efficacy for the control group being greater than that for the experimental group and the difference being greater than a futility boundary, then accrual of biomarker-negative patients stops; but the strategy continues with accruing additional biomarker-positive patients in order to substitute the unaccrued biomarker-negative patients until the pre-specified total target sample size is achieved; (iv) contrarily, if the interim results are promising in the biomarker-negative patients, the accrual of both biomarker-negative and biomarker-positive patients continues until the total target sample size is achieved.

**Methodology**: A pre-planned total sample size with futility stopping is considered for this two-stage adaptive design. The trial assesses the treatment effect both in the entire population and in the biomarker-positive population. Wang et al. (2007) [80] performed a simulation study testing a composite hypothesis; the hypothesis of the global treatment effect and a hypothesis of treatment effect in the biomarker-positive subgroup. A bivariate normal model which incorporates the correlation between the two test statistics for each hypothesis was used. Furthermore, two multiplicity adjustment methods which have a strong control of experimentwise false-positive rate (*α* = 0.025) were considered in order to test the composite hypotheses of no treatment effect; the first method was the equal split-alpha method which allocate *α*_1_ = *α*_2_ = 0.0125 [106] and the second method was the Hochberg’s method [107] for multiple testing; a special case of partitioning *α* which starts with the least significant p-value and investigate the other p-values in a sequential manner until it reaches the most significant one (unequal alpha split).

**Statistical/practical considerations**: Although a greater power is achieved as compared to a non-adaptive trial design in simulation settings, this strategy can yield an important increase in the duration of recruitment depending on the prevalence of the biomarker. Additionally, it does not allow for early termination of the study and can lead to biased treatment effect estimates when the results from interim analysis are used for selection or exclusion of a biomarker-defined subgroup. In addition, this study design is appropriate when there is rapid outcome assessment relative to the accrual rate and assumes complete confidence in the biomarker at the outset. A further limitation is that the futility boundary is considered conservative and less than optimal.

#### Adaptive parallel Simon two-stage design

Jones and Holmgren (2007) [85] proposed a Phase II adaptive design (Fig 6) by extending the Simon two-stage design [88]. This strategy does not include a control arm yet, consequently it can be considered a single-arm approach exactly like the Simon two-stage approach. The biomarker-adaptive parallel Simon two-stage design was mentioned in 8 (7.5%) papers of our review. The design aims to test a novel treatment which possibly has a different treatment effect in the biomarker-positive versus the biomarker-negative subgroups. This approach requires a pre-defined biomarker with well-established prevalence and permits preliminary determination of efficacy that may be restricted to a particular subset of patients. An example of actual trial which uses this strategy is the NCT00958971 trial [76, 92, 108].

**Design**: The design begins with two parallel phase II studies. During the first stage, two separate studies are performed in the biomarker-positive and biomarker-negative subgroups. Next, depending on the interim results of the first stage, the trial either stops or continues into a second stage with the enrollment from either the entire patient population (unselected patients) or from the biomarker-positive subpopulation only (selected patients). If a preliminary efficacy is observed during the first stage of the study for the experimental treatment in both the biomarker-positive and biomarker-negative subset, then additional patients from the general patient population will be enrolled in the second stage; if the interim result during the first stage of the trial shows that the efficacy is limited to the biomarker-positive subjects, then the recruitment of additional biomarker-positive patients only continues during the second stage.

**Methodology**: If there are sufficient results in both first and second stages, the novel treatment can further be explored. More precisely, the strategy is as follows as outlined by McShane et al. (2009) [90]: In the first stage of the design, $N_{1}^{-}\;$ biomarker-negative individuals and $N_{1}^{+}\;$ biomarker-positive individuals are recruited. An interim analysis is undertaken with its results determining how the design proceeds as follows: If the number of responses to the novel treatment in the biomarker-negative group, in the first stage $\;X_{1}^{-}$, meets or exceeds a cutoff of ${\;k}_{1}^{-}$, then *N*^*un*^ additional unselected individuals are accrued during the second stage (including $X_{2}^{-}$ biomarker-negative responders and $X_{1}^{+}\;$ biomarker-positive responders). If $X_{1}^{-}$ is less than $k_{1}^{-}$ but the number of responses in the biomarker-positive group in the first stage,${\;X}_{1}^{+}$, meets or exceeds a cutoff of $k_{1}^{+}$, then the design enrolls $N_{2}^{+}$, additional biomarker-positive individuals during the second stage (including $X_{2}^{+}\;$ responders). If $X_{1}^{-}$ is less than $k_{1}^{-}$ and $X_{1}^{+}$ is less than $k_{1}^{+}$ then the trial stops. Consequently, when the second stage is terminated, a total of *N*^+^ and *N*^−^ biomarker-positive and biomarker-negative individuals, respectively, will have been enrolled, whilst a total of $X_{T}^{+}$ (biomarker-positive group) and $X_{T}^{-}$ (biomarker-negative group) responders will have been observed.

At the end of stage two, treatment benefit is determined as follows: In the case where unselected individuals continued to be accrued during the second stage, the total number of responders in the biomarker-negative subgroup, $X_{T}^{-}$, is compared to a cutoff, *k*^−^ whilst the total number of responders in the biomarker-positive subgroup, $X_{T}^{+}$, is compared to a cutoff, *k*^+^. If ${\;X}_{T}^{-}\geq {\;k}^{-}$, then we conclude that the experimental treatment is beneficial in the unselected population; if $X_{T}^{+}\geq {\;k}^{+}$ and $X_{T}^{-}<{\;k}^{-}$ then we conclude that the treatment is beneficial only in the biomarker-positive population; if $X_{T}^{+}<{\;k}^{+}\;$ and $X_{T}^{-}<{\;k}^{-}$, then we conclude no treatment benefit. In the case where only biomarker-positive patients continued to be accrued during the second stage, $X_{T}^{+}$, is compared to a cutoff, $k_{2}^{+}$. If $X_{T}^{+}\geq k_{2}^{+}$ then we conclude treatment is beneficial in the biomarker-positive subgroup; otherwise we conclude no treatment benefit. The trial stage- and subgroup-specific sample sizes $\;{\;N}_{1}^{-}$,$\;N_{1}^{+}$, *N*^*un*^,$\;N_{2}^{+}$ and cutoffs $k_{1}^{-}$, $k_{1}^{+}$, *k*^−^, *k*^+^, $k_{2}^{+}$ are determined so that they control the probability of correct conclusions in the biomarker-positive and unselected patient groups.

Jones and Holmgren (2007) [85] have used the values 34, 34, 32 and 36 for $N_{1}^{-}$,$\;N_{1}^{+}$, *N*^*un*^, and $N_{2}^{+}$ respectively and the values 2, 1, 4, 4 and 5 for $k_{1}^{-}$, $k_{1}^{+}$, *k*^−^, *k*^+^ and $k_{2}^{+}$ respectively. As stated by Jones and Holmgren (2007) [85] values for the cutoffs $k_{1}^{-}$ and $k_{1}^{+}$ (equal to 2 and 1 respectively) are obtained from the first stage of the optimal Simon two-stage design. Additionally, in the case where there is preliminary efficacy of the experimental treatment in the unselected population during the first stage of the trial, the study enters the second stage where the values of *k*^−^ and *k*^+^ for decision making need to be defined. Assuming the total number of biomarker-positive subjects (*N*^+^) enrolled by the end of the second stage is fixed at its expected value given a known prevalence, the aforementioned values (*k*^−^ and *k*^+^) can be acquired as the minimum values needed for exclusion of the null value from the (1 − *α*) × 100% exact Blythe-Still-Casella confidence interval where *α*≤0.05; these values can be found using the StatXact software package. However, if the observed total number of biomarker-positive subjects is much different from the expected value, then the cut-offs (*k*^−^ and *k*^+^) can be changed using the confidence interval approach aiming to preserve the desired operating features of the design. Moreover, the value of $k_{2}^{+}$ needed also during the second stage of the trial for decision making can be acquired using either the confidence interval approach or through the calculation of exact binomial probabilities.

**Statistical/practical considerations**: The Adaptive Parallel Simon two-stage design may be considered as a simple approach with reasonable operating characteristics which can result in sample size savings as compared to the Simon two-stage design [88], however, one major drawback is that early termination of the study is not allowed during the initial stage of the trial for efficacy in a single biomarker-defined subgroup. Additionally, this approach requires the pre-specification of appropriate response rates in both biomarker-positive and biomarker-negative subgroups which may be difficult.

#### Multi-arm multi-stage designs

Multi-arm multi-stage (MAMS) designs were found in 16 (14.9%) papers. They have the ability to simultaneously compare multiple experimental treatments with the standard treatment in order to achieve more reliable results in less time as compared with separate Phase II trials to assess each novel treatment individually. An intermediate outcome measure is used to identify both treatments for which there is an early sign of effectiveness and treatments that appear ineffective thus allowing the study to continue with the promising experimental arms and to stop the investigation of insufficient treatments. Generally, MAMS designs, according to Parmar et al. (2008) [97], are useful when (i) there are multiple promising treatments in phase II/III studies; (ii) there is no strong belief that a treatment will be more beneficial compared to another therapy; (iii) availability of adequate funds; (iv) there is an adequate number of patients to be enrolled and (v) there is an intermediate outcome measure correlated with the primary outcome measure. Parmar et al. (2008) [97] encouraged the use of the MAMS strategy in the field of oncology but highlighted that these designs should only be used when quick outcome assessment is possible [69]. There are two variants referred to as i) Two-stage adaptive seamless design, ii) Group Sequential design to the MAMS designs with differences occurring in its methodology. Information about these variants can be found in S1 File, section ‘‘Variations of Multi-arm multi-stage (MAMS) design”. Some examples of actual trials which use the MAMS approach are the following: i) GOG-182 [20, 97, 102], ii) STAMPEDE [93, 97], iii) ICON6 [93, 97, 109], iv) FOCUS4 trial [69, 103].

**Design**: Fig 7 illustrates a MAMS design where the first stage of the trial (the Phase II stage) involves randomization within one of two arms which simultaneously compare two experimental treatments with the standard of care (control) using an intermediate outcome measure (e.g. progression free survival). The arm within which a patient is included depends on their biomarker status, for example patients positive for biomarker 1 may be randomized in arm 1 to either standard of care or experimental treatment 1 whilst patients positive for biomarker 2 may be randomized in arm 2 to either standard of care or experimental treatment 2. At the end of this first stage, an interim analysis is undertaken in each arm, comparing the experimental treatment with standard of care. Depending on the outcome of the interim analysis, accrual of patients either continues within an arm to the second stage of the trial or the accrual of additional patients stops within that arm. Despite the fact that some experimental treatments cannot pass the first stage, a secondary analysis can be conducted for each of these treatment arms comparing them with the standard of care. This approach ensures that patients are randomized to the most promising treatments which were selected at the first stage of the study.

**Methodology**: At the interim stage, in the case where the observed effect size in an experimental arm is greater than a predefined critical value, accrual of patients continues within that arm to the second stage of the trial until the pre-specified number of events on the primary outcome (e.g. overall survival) measure is reached, otherwise the accrual of additional patients stops within that arm and the corresponding novel treatment does not enter the second stage of the trial. The aforementioned predefined critical value is calculated for each stage of the study in a way that takes into account whether the null hypothesis can be rejected at the level of the probability of the continuation of the study to the next stage should the null hypothesis be true as Parmar et al. (2008) [97] state.

The stopping thresholds are based on test statistics, resulting in dropping experimental arms which do not show effectiveness. The allocation to each remaining arm is fixed in MAMS trials, however, it is possible to assign more patients in the control treatment group than to the experimental arms which can yield small gains in efficiency over balanced randomization as Wason and Trippa (2014) [69] highlighted; this strategy has been used in practice with the STAMPEDE trial where the control arm is compared with five experimental treatments with the corresponding randomization ratio 2:1:1:1:1:1 [93]. MAMS approach could be designed with either a fixed sample size by fixing the number of patients enrolled at each stage or a fixed number of patients enrolled per arm per stage [69].

The methodology has mainly focused on situations where the primary endpoint is assumed normally distributed or time-to-event [69]. Two papers discuss MAMS designs with the normally distributed endpoint [94, 110], whilst a time-to-event endpoint is used by Royston et al. (2003) [99]. Freely-available software in Stata for calculating sample size was proposed by Barthel et al. (2009) [97] for MAMS trials [93].

A recent article by Wason *et al*. (2015) [111] proposed a new Bayesian adaptive design for clinical trials with biomarkers and linked treatments in multi-arm phase II trials. It is a novel approach combining the methodology used for BATTLE, I-SPY 2 and FOCUS 4 trial, which results in significant power to identify treatment effects. This novel trial design could be used for simultaneously testing several predictive biomarkers and new experimental treatments in a more cost-effective and rapid way.

**Statistical/practical considerations**: MAMS designs as compared with testing each experimental treatment in separate large-scale two-armed trials not only shorten the length of time required and reduce the costs due to the fact that they assess several experimental treatments at the same time while using a smaller number of individuals as some experimental treatment arms are dropped early. Despite the aforementioned benefits, researchers are faced with operational challenges and difficulties in building-up such designs.

#### Stratified adaptive design

Tournoux-Facon et al. (2011) [89] proposed a new Adaptive Stratified phase II design based on the multiple-stage Fleming design [112]. A single article (0.93%) of our review referred to this approach. It is an alternative approach to dealing with stratification in a phase II setting and aims to demonstrate whether an experimental treatment (a control arm is not included, thus it’s about a single arm approach) is beneficial for at least one biomarker-defined subgroup rather than the entire study population.

**Design**: An illustration of this approach is given in Fig 8. The first stage is consisted of an interim analysis where the response rate is estimated in the biomarker positive and biomarker negative subgroups separately. The trial then enters a second stage and depending on the results of the interim assessment, accrual continues either from the entire patient population if there is treatment efficacy of both biomarker-defined subgroups, or from one of the distinct biomarker subpopulations only in which treatment efficacy has been observed.

**Methodology**: Decision making and the number of patients used at the second stage of the trial are based on the observed response rates during the first stage of the trial. This approach depends on the identification of heterogeneity between the two biomarker-defined subgroups (positive and negative subgroups). Heterogeneity is identified when the observed response rate in one of the biomarker-defined subgroups is less than *π*_0i_ (defined as the probability of response in one of the biomarker-defined subsets below which the novel treatment is considered to be a low-activity treatment, where *i* denotes each biomarker-defined subgroup; the value of 0.25 is used for the *π*_0i_ by Tournoux-Facon et al. (2011) [89]), whereas the other subset has a response rate greater than *π*_0i_. The subset for which the observed response rate is less than *π*_0i_ is considered clinically insignificant, and therefore cannot continue to the second stage of the trial. Only the subgroup with response rate greater than *π*_0i_ therefore enters the second stage where the study can continue as a randomized Phase III trial comparing the novel treatment which has proved to be effective with the standard of care. More precisely, the identification of heterogeneity of responses is performed by calculating the symmetric interval of probability around *π*_0i_ at each stage (only a symmetric interval is observed due to binomial calculation). When the first stage of the design is terminated, in case that the cumulative number of responses for one of the biomarker-defined subset is less than/greater than the lower/upper boundary of the aforementioned symmetric interval of probability and the cumulative number of responses for the other biomarker-defined subgroup is greater than/less than the upper/lower boundary of the symmetric interval, then the responses between the two subsets are considered heterogeneous; otherwise, the treatment effect is similar in the two subsets, consequently, the trial continues without selecting any biomarker-defined subset. After the identification of heterogeneity of responses, conclusions at the end of the first stage of the trial are made according to decision rules based on specific thresholds which are determined by iterations using a Fleming two-stage approach [112]; a single-arm design which permits early termination of the trial for either efficacy or inefficacy of the treatment.

The adaptive stratified design has a number of differences from the Adaptive Parallel Simon two-stage design proposed by Jones and Holmgren (2007) [85] and the global one-sample test for response rates for stratified phase II clinical trials proposed by London and Chang (2005) [113]. First and foremost, the adaptive stratified design permits early stopping for inefficacy or efficacy of the study as it is a strategy based on a Fleming design [112]. On the contrary, the two aforementioned methods are based on the Simon design and do not make the discontinuation of the study possible. Additionally, the stratification approach used in the design provided by Tournoux-Facon et al. (2011) [89] is utilized in order to target the patients who are most likely to respond to a novel treatment, whereas, stratification in the design by London and Chang (2005) [113] aims to ameliorate the power of the overall test.

**Statistical/practical considerations**: Tournoux-Facon et al. (2011) [89] state several benefits, such as the possibility of early termination for efficacy or inefficacy of the novel treatment according to the results of the interim analysis (first stage). Moreover, this approach can be considered more ethical due to the fact that it identifies a particular biomarker-defined subpopulation for which the novel treatment can be effective and thus avoids conducting a study with patients exposed to toxic treatments. Additionally, this strategy ameliorates targeting of the populations entering phase III trials. No statistical challenges have been identified for this type of trial design so far.

#### Tandem two stage design

The tandem two-stage design was discussed in 5 (4.7%) papers. It was proposed by Pusztai et al. (2007) [104] and it is composed of 2 optimal trials in a Phase II settings (Fig 9). This design was proposed for rapid biomarker assessment in settings where we don’t know the activity of a novel treatment in the unselected population but there is at least one candidate predictor of response. This approach can identify whether the novel treatment is effective in the unselected patients, and if it is not, can tell us if the predictor can enrich the responding population [104]. Only an experimental treatment arm is included in this design and not a control treatment arm, thus this approach can be considered a single-arm approach. An example of actual trial which uses the tandem two stage approach is the NCT00735917 [90, 92, 114, 115].

**Design**: In this design, a predefined biomarker is assumed. In the first stage of the trial, patients from the entire population enter the trial irrespective of their biomarker status. An interim analysis is then undertaken and if a sufficient number of events (defined in terms of clinical benefit rate or response rate) have been observed during the first stage, the study proceeds to a second stage whereby further patients are accrued from the unselected population to establish the benefit rate more precisely in unselected patients. However, if an insufficient number of events have been observed during the first stage, rather than stopping accrual for futility, a second trial commences whereby its first stage involves continued accrual of biomarker—positive patients only. An interim analysis is then conducted and if a sufficient number of events have been occurred, this second trial continues into a second stage of biomarker-marker positive patient accrual. Otherwise, if an insufficient number of events have occurred, the predefined biomarker is rejected.

**Methodology**: A second phase in the trial design is considered due to the fact that the small number of individuals used in the first phase of the study (typically n1<25) is likely to include insufficient number of biomarker-positive individuals in order to decide whether the novel treatment benefits this particular biomarker-defined subset. In terms of defining what constitutes a ‘sufficient number’, Pusztai et al. (2007) [104] suggest the use of a noninformative prior distribution for clinical benefit rate of *β*(1,1) and make recommendations for the early stopping rules. More precisely, Pusztai et al. (2007) [104], given a certain value for the targeted level of activity of the novel treatment (i.e., 25% clinical benefit rate), suggest that the trial should stop early for futility if the conditional power (i.e., the chance to reach the aforementioned targeted level of activity) is equal or less than 7.5% in the following cases: (i) at the first 9 evaluated patients there is no one who responds to treatment; (ii) at the first 15 evaluated patients there is only one individual who responds to treatment and (iii) at the first 20 evaluated patients there are only 2 individuals who respond to treatment.

The sample size for this approach is calculated with the same rules as a classic two-stage or Bayesian phase II design [104] where criteria for specifying the sample size are used (e.g. one criterion is to choose a sample size so that if there is no early termination of the trial and the trial accrues the entire population the posterior of the experimental treatment success rate reaches a specified degree of precision). The sample size calculations are discussed in two papers [116, 117].

**Statistical considerations**: The two trials within this design could be conducted separately, as two independent trials for the unselected individuals and for the biomarker-positive individuals, however, this can result in larger duration and costs, therefore it would be better to run the two trials as a single study (see Table 1 for further details). Additionally, this approach enables the estimation of response rates in both biomarker-negative and biomarker-positive patients.

## Discussion

The review has demonstrated ambiguity and confusion regarding biomarker-guided adaptive designs proposed by different authors. In this review, we focus on 8 types of such designs. There are several reasons why these design strategies are becoming an appealing approach to a great extent. The main reason is their application to real clinical practice and their ability to evaluate both multiple experimental treatments and biomarkers simultaneously. Hence, multiple questions can be answered just in a single trial [48]. During the progression of the trial alterations are permitted, and consequently, any potential incorrect hypothesis made at the beginning of the trial can be modified. Many authors note that these strategies are ethical in terms of safety and efficacy as they attempt to tailor the appropriate treatment to the right population at the right time [10, 33, 37, 40, 46, 55, 70, 118, 119]. The required number of patients needed for the enrollment in the trial can be modified according to the results from interim analysis (e.g. stop accrual or increase sample size) and the duration of the trial can be minimized as they allow for dropping early treatments which show poor performance. Also, due to alterations, e.g. if incrementation of the sample size is suggested as the study progresses, higher power to demonstrate a treatment effect may be achieved [120]. Furthermore, it has been argued that during the adaptation process, preservation of type I and type II error rates may be attained through the appropriate choice of statistical parameters [26].

Despite the aforementioned advantages, there are a considerable number of challenges which should be carefully investigated before making a decision. Their implementation may be considered a poor choice when there is already a high quality retrospective dataset available which includes information both on biomarker status and on long-term follow-up, since in such a situation an analysis of this dataset to identify a biomarker subgroup would likely be more efficient as a first stage as opposed to incorporating this first stage into the trial itself [120]. Also, they can be complex in terms of logistic issues such as maintaining trial integrity, minimising operational bias [33, 45, 48, 52] and the involved perspectives of regulatory agencies (e.g. what level of adaptation will be acceptable to the regulatory agencies) [121]. In addition, adaptations, of which statistical validity may be challenging, can lead to notable modifications yielding a complicated trial totally different from the initial study [33, 37, 40]. Consequently, it could diverge from the original question which researchers expect to answer. Furthermore, statistical validity of conclusions can be influenced to a great extent as unexpected bias or variation may be introduced during the course of the trial making the interpretation of results greatly complex [33, 122, 123]. The inserted operational bias occurred by the modifications in the trial design augments the likelihood of making a false conclusion that the treatment is beneficial to certain patients [33, 37, 40, 71, 118]. It is necessary that adaptive designs are planned in such a way that allows for controlling both Type I and Type II error rates [69]. Additionally, from a statistical viewpoint, adaptive designs based on Bayesian methods are considered computationally intensive [55] and estimations of Type I error rate can be inaccurate. Problems of statistical testing may also arise and applying the statistical methods can be very challenging without the availability of appropriate software packages to facilitate the implementation of adaptive designs (e.g. computational intensive demands of Bayesian methods) [33, 40, 45, 48, 52]. A number of obstacles and barriers related to the conduct of adaptive designs in practice in Phase III trials is addressed in a recent paper [124]; several key stakeholders in clinical trials research have been interviewed and some of the highlighted difficulties expressed during this study were the lack of appropriate knowledge and familiarity of these designs in the biostatistics community, insufficient time and funding structure, additional work required due to the complexity of such designs and the needed statistical expertise and appropriate software.

However, adaptive designs will continue to hold a prominent place in the era of personalized medicine, and hence, further developments and discussion are of utmost importance in order to enhance clinical research. In conducting such further developments and discussion, investigators should take account of the following points in particular (i) regulatory and logistical issues; (ii) statistical challenges including the control of the false-positive rate, power of the study and treatment effect estimation; (iii) the unexpected bias likely to be introduced during the adaptation process and (iv) the potential increased cost and time. Further, the different designs proposed so far for adaptive trials need to be better understood by the research community, as the proper use of such designs can result in a great increase in the efficiency of a trial and boost the development of novel treatments. By conducting this methodological review, we contribute to the knowledge enhancement of researchers regarding the biomarker-guided adaptive trial designs.

The characteristics and methodology of the eight main designs are discussed in the current paper, whilst information on their variations are summarized in S1 File (Table A in S1 File). Additional references for these variations are provided in [125–142].

## Supporting Information

S1 File Variations of biomarker-guided adaptive trial designs. (DOCX)

S1 Keywords Literature review search strategies for both biomarker-guided clinical trial designs and for traditional trial designs. (DOCX)
