# Supplementary material for: Biomarker-Guided Adaptive Trial Designs in Phase II and Phase III: A Methodological Review
Source: PLoS One. 2016 Feb 24;11(2):e0149803. doi: 10.1371/journal.pone.0149803 (PMC4766245; doi:10.1371/journal.pone.0149803)
Supplement: S1 File — (DOCX) [file pone.0149803.s001.docx]

**S1 File.** **Variations of biomarker-guided adaptive trial designs**

The variations of the main biomarker-guided adaptive designs are discussed below and characteristics are summarized further in Table A.

***Variations of Adaptive signature design***

**Adaptive threshold design**

This variation of the aforementioned Adaptive Signature design is mentioned in 25 papers (23.4%) of our review. The difference between the Adaptive Signature design and the Adaptive Threshold design is that the first one is used to develop and validate a biomarker, whereas this variant tries to identify and validate an optimal cut-off point for a pre-specified biomarker. In other words, the Adaptive Threshold design was suggested for settings in which a putative biomarker is measured on a continuous or graded scale with its threshold for detecting individuals who would benefit from the novel treatment not predefined at the initial stage of a Phase III trial. In terms of Fig 2, the difference between the main design (Adaptive Signature design) and this variant corresponds to the biomarker-positive subset. More precisely, in the main design, if there is no claim of treatment effectiveness in the entire population, then a portion of individuals is used to develop a predictive biomarker signature and the remaining portion is used to compare the treatment effect. However, in this variant if there is no claim of treatment effectiveness in the entire population, the design identifies and validates a cut-off point for a prospectively selected biomarker. Adaptations here are referred to the subgroup and there are no modifications regarding the required number of patients or randomization ratio. In this design, human samples are collected to measure a pre-specified biomarker from the entire population at the beginning of the study but the value of biomarker is not used as an eligibility criteria.

Two analysis plans compose this approach, the so-called ‘analysis plan A’ and ‘analysis plan B’. The first plan is identical to the strategy proposed for the Adaptive Signature design. The second plan uses a more effective method to accommodate the multiplicity issue when combining the statistical tests for the entire population and the biomarker-defined subgroup by incorporating the correlation structure of the two test statistics. More precisely, the plans A and B are different in the way the test statistics and thus its distribution assuming true null hypothesis is calculated. For Plan A, the test statistic is calculated as the maximum, across all possible cutoff values, of the log-likelihood ratio statistic for treatment effect for those with biomarker values above the cutoff value. For plan B, the test statistic is calculated as the larger of the test statistic for procedure A and the log-likelihood ratio statistic for treatment effect in the entire population. The second plan is considered a generalization of the first plan because in case that a difference between experimental treatment and the standard of care is demonstrated, then the next stage is to find the biomarker threshold above which the targeted treatment is more beneficial for patients than the control treatment, consequently, among the cut-off points of a measured score of a biomarker, the maximum value is selected. This plan uses a larger sample size, resulting in appropriate power for establishing the statistical significance of treatment effect restricted to patients with biomarker values above an initially unknown cut-off point.

Jiang et al*.* (2007) [125] proposed the use of bootstrap re-sampling method for the estimation of the point estimate and a confidence interval for the cut-off point and described the sample size planning for this design. Also, according to Jiang et al. (2007) [125] if plan B does not reject the null hypothesis, the estimation of the cut-off point value would be inexplicable, and thus it should not be estimated. Jiang et al. (2007) [125] demonstrated through a simulation study that the second plan was more effective than the first one. A modification of the global test of the null hypothesis which was used by Jiang et al. (2007) [125] is illustrated in the paper of Simon (2012) [57]. The Adaptive Threshold design can detect efficiently a global treatment effect and provides statistically valid tests when the promising treatment effect is limited to a particular biomarker-defined subset, however, a larger sample size may be required and can lead also to redundant power.

**Molecular signature design**

Molecular Signature design is mentioned in 2 two articles (1.9%). It is a Phase III design which collects tissue samples from the entire population at the start of the trial and analyse them when the study is near completion.

After the collection of tissue samples from the entire population, all patients are randomized to either the experimental treatment or the standard treatment. The methodology is similar to the Adaptive Signature design. This approach makes the comparison of the novel drug with the standard of care, but on a primary outcome measure which here is the overall survival using the significance level of 0.04. In case that the results show the effectiveness of an experimental treatment over the control arm, we claim the effectiveness of treatment in the overall population. Otherwise, an analysis is conducted for the identification and validation of the biomarker classifier (i.e., a combination of biomarkers) which gives the best primary outcome measure. A portion of subjects is used for the detection of a biomarker classifier and the remainder of patients for its validation. It is considered as a promising strategy without statistical considerations mentioned.

**Cross-validated Adaptive Signature design**

Cross-validated Adaptive Signature design (CVASD) is found in 19 papers (17.8%) of our review. It was proposed by Freidlin et al. (2010) [60] aiming to increase the efficiency of the Adaptive Signature design. Similar to the Adaptive signature approach it is a Phase III frequentist trial design based on a fall back strategy in order to identify candidate biomarkers in the training set of the study and evaluate them in the validation set.

The difference between Adaptive signature design and Cross-validated Adaptive Signature design is in terms of the methodology analysis. The former is composed of a split-sample approach, using approximately half of patients to develop the biomarker signature and the remainder of patients to validate it, whereas, the latter uses the K-fold cross validation procedure, i.e., there are K cross-validated training sets which are used to classify subjects in the corresponding K cross-validated validation sets. After the classification of all patients, we compare the experimental treatment versus the control treatment in the biomarker-positive patients (i.e., subgroup of classifier positive patients). The Cross-validated Adaptive Signature design may yield larger power but it faces the same challenges with its main design and also includes the multiplicity problem.

**Generalized adaptive signature design**

Generalized Adaptive Signature approach is described in 2 papers (1.9%). Firstly, candidate biomarkers are selected and the cut-off points are optimized using a training set and secondly, the chosen biomarkers are assessed in the validation set. According to Simon (2010) [18], this approach is applicable when there are a number of available candidate biomarkers, but data from a Phase III setting is required for choosing the most appropriate biomarkers. A major drawback of this design is the limited power when we assess the treatment effect in the biomarker-defined subset.

**Adaptive signature design with subgroup plots**

Adaptive Signature design with Subgroup Plots [64] is an extension of Adaptive Signature design which has been proposed in order to add flexibility. It uses tail-oriented or sliding window subgroup plots in order to identify a subset of patients which is most likely to respond to a particular experimental treatment after taking into account several cut-off points of the benefit score obtained by the subgroup plots. In this way it provides broader confidence intervals of the estimated treatment benefit. No statistical considerations have been found for this approach.

***Variation of Outcome-based adaptive randomization design***

**Bayesian covariate adjusted response-adaptive randomization**

The Bayesian Covariate Adjusted Response-Adaptive Randomization (BCARA) is identified in two articles (1.9%) and it was proposed in 2010 by Eickhoff et al. (2010) [53]. This strategy which combines a Bayesian, an adaptive and biomarker classification approach aims to match patients with the most efficacious treatments by utilizing patient’s biomarker information becoming available during the conduct of the clinical trial. This strategy may be useful in the explanatory phase II setting of the drug development [53]. It is also considered as a response-adaptive randomization strategy as the allocation of the study population depends on the responses of previous outcomes. A partial least square logistic regression approach is conducted to determine adaptively predictive biomarker-defined subsets.

The general procedure of this approach is composed of four steps according to Eickhoff et al*.* (2010) [53]: (i) randomly assign the first $n^{*}>=J^{*}(K+1)$ patients to the different treatment arms where J the number of different treatment groups and K the number of biomarkers. At least one response should be observed in each of the different treatment groups before moving to the Bayesian response adaptive randomization; (ii) after each new individual has been enrolled in the study, predictive biomarker-defined groups are determined by utilizing a partial least squares logistic regression strategy (PLSLR) which can predict whether the patient can benefit from the treatment. The biomarker status is determined before the randomization; (iii) after the establishment of the biomarker status and biomarker-defined groups of each new individual, the individual is then randomly assigned into one of the treatment arms using a BCARA randomization; (iv) according to the results of the BCARA randomization the trial either stops or continues based on decision rules proposed by Eickhoff et al. (2010) [53]. The Bayesian covariate adjusted response-adaptive trial design has the ability to identify the biomarker-defined groups likely to respond to a treatment but it does not control the Type I error and in order to ensure that the identified result is true, a Phase III study should be conducted.

***Variation of Adaptive patient enrichment design***

**Modified Bayesian version of the two-stage design of Wang et al. (2007) [80]**

A variation of Adaptive Patient Enrichment design by Wang et al. (2007) [80] was found in 2 papers (1.9 %). It is a Phase III Bayesian two-stage design proposed by Karuri and Simon (2012) [7] for the evaluation of both treatment and biomarker.

Karuri and Simon (2007) [7] use a Bayesian framework in order to allow further flexibility for expressing the degree of prior information regarding the utility of a biomarker. More precisely, posterior distribution of treatment effects within the biomarker-positive and biomarker-negative subgroups based on an interim analysis of first-stage is used in order to come to a decision regarding the recruitment and the continuation of the trial. This approach allows for early termination of the study during the initial stage of the trial and has a satisfactory power. No statistical challenges have been identified.

***Variations of Multi-arm multi-stage (MAMS) design***

**Two-stage adaptive seamless design**

Two-stage Adaptive Seamless design is a type of clinical trial design identified in 28 papers (26.2%) of our review. It uses the MAMS approach combining two separate studies into one single study and uses interim monitoring as well as multi-arm design features. It connects the explanatory Phase II stage for treatment selection and confirmatory Phase III stage for the final comparison of the chosen experimental treatments with the standard of care. The Two-stage Adaptive Seamless design aims to improve the power in the Phase II stage in order to continue on the Phase III stage having obtained important promising information. In the definitive analysis, it uses data from patients registered during the Phase II and Phase III stages. A prerequisite of this strategy is the availability of a reliable early endpoint. An example of actual trial which uses the two-stage adaptive seamless design is the ISPY2 trial [54, 93, 126, 127].

Brannath et al. (2009) [36] propose an approach which uses Bayesian decision tools and is based on the two-stage seamless design in order to confirm that the identified biomarker-defined subgroup from a Phase II study is sensitive to the new treatment in a separate explanatory phase (i.e., a study which is conducted at the same time with the two-stage adaptive seamless design) and afterward conduct a Phase III study with this selected subgroup. More precisely, the general procedure of this Phase II/III strategy is presented by Brannath et al. (2009) [36] as follows: When half of individuals are recruited in the study, an interim analysis is performed in order to decide whether to accept or not a biomarker-defined subpopulation identified in a separate exploratory study. At this interim stage, a decision is also made about whether to continue accruing patients from the aforementioned biomarker-defined subset or from the entire study population. If the first case occurs, the treatment effect is assessed only in this biomarker subpopulation and if the second case happens, the treatment effect is tested in the entire population and biomarker-defined subgroup at the same time. In case that there is no identified biomarker-defined subpopulation from the separate exploratory study, the trial continues in the overall population using a classical group sequential design. The major advantage of this type of design is its ability to reduce the costs and also the selection of the target population in a reliable way. Also, appropriate methodology, such as that used by Brannath et al. (2009) [36] where multiple testing is adjusted by a weighted combination of p-value from data of the second stage and the first stage, and Simes’ step-up process [128] is used when combining data form both Phases in order to maintain the Type I error rate. An extension of the above approach by Brannath et al. (2009) [36] is proposed by Jenkins et al. (2011) [129] which can result in the rapid approval of novel treatments to the most appropriate individuals who are likely to benefit from the new drug. During the Phase II trial an interim analysis is conducted using a short-term intermediate outcome measure (i.e., survival endpoint) in order to select the population (either the entire population or the biomarker-positive patients) which will be used in the Phase III study with a long–term endpoint.

Mehta et al. (2014) [130] proposed an alternative seamless approach for subgroup selection in time-to-event-data for situations where there is no *a priory* assumption that a biomarker is predictive of treatment efficacy; consequently their design tests whether there is treatment effect in both biomarker-negative and biomarker-positive subpopulation separately instead of testing the null hypothesis of no treatment effect in the entire study population and in biomarker-positive subset.

According to Scher et al. (2011) [59], formulas for sample size calculation/allocation are proposed in situations where the study endpoints are continuous, discrete, and contain time-to-event data supposing the availability of a well-established relationship between the study endpoints at different stages, and that the study objectives at different stages are the same. Ang et al. (2010) [52] have stated that even in case that the trial stops early, a Phase III infrastructure should be developed. Such strategies have been proposed by Ellenberg and Eisenberger (1985) [131] and Inoue et al. (2002) [132] for evaluating the possibility to stop early or to continue to the confirmatory phase III repeatedly during the explanatory phase. The aforementioned designs are useful in situations where there is strong belief in the efficacy of the experimental therapy that can lead the study to the confirmatory phase, but confirm of this assumption is needed [52]. Despite the fact that this approach is considered as a more efficient strategy yielding larger power as compared with the conduct of separate trials, it can lead to introduction of bias and inflation of the type I error rate.

**Group Sequential design**

Adaptive Group Sequential design is found in 2 papers (1.9%) which can be incorporated into the MAMS approach for the development and validation of personalized therapies and is proposed by Lai et al. (2013) [74]. This strategy aims to find the most beneficial treatment for future patients based on their biomarker profiles, with a guaranteed probability of correct selection. It was proposed for the examination of multiple composite hypotheses not only in the entire study population but also in the biomarker-positive subgroups [133].The design is based on approved treatments, and aims to improve patient’s health by providing them with the most efficacious (yet unidentified) treatment. Additionally, another crucial objective of this approach is that the development of a novel treatment strategy for the forthcoming patients and the confirmation that the treatment effect of this strategy is in fact more effective than the historical mean effect of the control treatment plus a predetermined threshold [74].

According to Lai et al. (2013) [74], it is an approach for “jointly developing and testing treatment recommendations for biomarker classes, while using multi-armed bandit ideas to provide sequentially optimizing treatments to patients in the trial”. According to an interim data analysis, sequential decisions about whether to continue the study or not, are taken. It is considered a simple approach where selection of cut-off points is not required before the conduct of the first interim analysis.

**Table A. Characteristics of variations of Biomarker-guided adaptive trial designs**

| **Types of variations of Biomarker-guided adaptive trial designs** | **Phase** | **Pros** | **Cons** |
| --- | --- | --- | --- |
| **Adaptive threshold design**  (25 papers)  [3, 6, 8, 12, 14, 15, 18, 20, 21, 27, 29, 30, 47, 57, 58, 63, 64, 68, 70, 74, 78, 84, 125, 134]  **Also called:**  Biomarker adaptive threshold design | III | Validation of a candidate biomarker without need for an established cut-off point.  Identification of an optimal cut-off point for detecting sensitive patients (i.e., biomarker-positive patients).  Detection of overall treatment effect if one exists.  Statistically valid test if treatment benefit is restricted to a biomarker-defined subgroup.  Reduces dependence on Phase II data for establishing a test cut-off point.  More efficient design as compared to the traditional design (i.e., standard broad eligibility Phase III design based on assessing the global treatment effect in the overall population when the proportion of sensitive patients is low). | Requirement of a pre-specified biomarker for sensitivity, but not an established cut-off point.  Data from the same study to both define and validate the cut-off point of the biomarker may raise concerns.  Augmented costs due to the potential sample size increase and/or redundant power by partitioning the overall type I error. |
| **Molecular signature design**  (2 papers)  [32, 63]  No alternative names found for this trial design | III | Considered as a promising strategy for drug development as it takes advantage of the use of an end-point with clear clinical gain. | No information found |
| **Cross-validated adaptive signature design**  (19 papers)  [9, 14-16, 18, 20, 21, 24, 27, 32, 59, 60, 62-64, 66, 84, 104, 135]  No alternative names found for this trial design | III | Gain more power as it could maximize the number of individuals taking part in the development of the biomarker signature.  Can detect the subset of patients most likely to respond to a specific treatment in a more reliable way. | Same challenges as the Adaptive signature design.  Multiplicity problem for statistical testing as the statistical test would be conducted twice. |
| **Generalized adaptive signature**(2 papers)  [18, 63]  No alternative names found for this trial design | III | Optimizes the test based on randomized data for patients in the Phase III setting. | Limits its power when testing the effectiveness of an experimental treatment in the biomarker-positive subgroup. |
| **Adaptive signature design with subgroup plots**  (1 paper)  [64]  No alternative names found for this trial design | III | No information found | No information found |
| **Bayesian covariate adjusted response-adaptive randomization**  (2 papers)  [53, 63]  No alternative names found for this trial design | II | Ability to incorporate prior knowledge from biomarkers into the design.  Identification of the subgroups for which a particular experimental treatment is more effective.  Can result in reduction of the number of patients required when compared to alternative designs (i.e, non-adaptive trial designs).  Solves the issue of the incorporation of information of multiple and possibly correlated biomarkers. | The Type I error is not controlled in the traditional sense.  An independent Phase III study focused on the selected biomarker-defined subgroups is required to show that the identified promising result is definitely true. |
| **Modified Bayesian version of the two-stage design of Wang et al. (2007) [80]**  (2 papers)  [7, 136]  **Also called:**  Two-Stage Bayesian design | III | Can incorporate prior belief regarding the strength of biomarker into the Phase III setting using a Bayesian framework and simultaneously protecting the study population and minimizing the Type I error in the biomarker-positive and biomarker-negative subgroups  Can terminate the study early according to whether the treatment is effective or not in the biomarker-positive subgroup at the interim stage whereas the main design by Wang et al. (2007) [80] does not allow for early termination of the trial.  Satisfactory power for testing the biomarker-positive subgroup.  Enables the reduction of number of biomarker-negative patients for whom a particular treatment tailored to them seems to be ineffective according to biological evidence.  The utilized Bayesian formulation sheds light on the nature of inference at the end of the study.  Can result in reduction of costs of clinical development. | No information found |
| **Two-stage adaptive seamless design**  (28 papers)  [20, 23, 33, 36, 37, 40, 42, 43, 45-47, 52, 54, 59, 68, 74, 82, 93, 119, 126, 129-131, 137-142]  **Also called:**  Seamless Phase II/III designs  Adaptive Seamless  Phase II/III Adaptive design  Two-stage Adaptive Seamless design  Adaptive Seamless Phase II/III design | II/III | The evaluation of each experimental therapy can be performed without requiring the conduct of separate large-scale Phase II trials.  Flexibility and efficiency of trials can be increased.  Individuals from both explanatory and confirmatory stages are used in the definitive analysis; hence, the design avoids ‘wasting’ individuals already registered in Phase II setting.  Diminishes the potential loss of time between the completion of Phase II stage and the beginning of patient enrollment in Phase III setting.  The same standard of care can be used in both stages of the study.  Can result in the same quality of evidence as in a traditional design but with a smaller number of patients.  Can result in the speedup of drug development and also in a successful Phase III trial.  More efficient due to the improved power and the ability to control the Type I error as compared with the conduct of separate studies.  The required power of both individual studies may be acquired by using a smaller number of patients than that of a single study.  Two separate trials (Phase II and Phase III) are conducted under one single trial protocol, but in reality, researchers analyze them separately using data from each stage, resulting in this way in savings of time and cost. | A significant concern is that the results obtained from the Phase II analysis, which becomes an interim analysis of the Phase III study should remain in the hands of the data monitoring committee due to confidentiality.  Concerns also arise regarding the efficiency and validity of such a trial design.  Sometimes, the endpoints within the different phases are dissimilar, hence, a decision is required on how to combine the data obtained from both stages in order to use them in the definitive analysis. This challenge is further discussed in the paper of Chow et al. (2007) [141].  According to the Draft Guidance by U.S. Food and Drug Administration (2010) [142], an adaptive seamless phase II/III design is described as a less well-understood design which may introduce bias and inflation of the Type I error rate.  Calculation and allocation of the necessary sample size for the two separate studies. |
| **Group Sequential design**  (2 papers)  [74, 133]  No alternative names found for this trial design |  | Researchers do not have to choose the cut-off points which should be used to designate the biomarker classes until the performance of the first interim analysis.  Non-promising treatments can be dropped at an early stage. | No information found |
