# Supplementary material for: Biomarker-Guided Adaptive Trial Designs in Phase II and Phase III: A Methodological Review
Source: PLoS One. 2016 Feb 24;11(2):e0149803. doi: 10.1371/journal.pone.0149803 (PMC4766245; doi:10.1371/journal.pone.0149803)
Supplement: S1 Keywords — (DOCX) [file pone.0149803.s002.docx]

**S1 Keywords. Literature review search strategies for both biomarker-guided clinical trial designs and for traditional trial designs.**

***MEDLINE***

**Traditional clinical trial designs**

1. Clinical Trials as Topic/

2. Clinical Trial/

3. 1 or 2

4. Research Design

5. design*.mp.

6. Research design*.mp.

7. Statistical design*.mp.

8. Study design*.mp.

9. Traditional design*.mp.

10. Trial design*.mp.

11. 4 or 5 or 6 or 7 or 8 or 9 or 10

12. 3 and 11

13. limit 12 to (english language and “review articles”)

14. limit 13 to last ten years

***MEDLINE***

**Biomarker-guided clinical trial designs**

1. Clinical Trials as Topic/

2. clinical trial*. ti, ab.

3. 1 or 2

4. design*. ti, ab.

5. 3 and 4

6. limit 5 to comment

7. limit 5 to editorial

8. limit 5 to journal article

9. limit 5 to guideline

10. limit 5 to systematic reviews

11. limit 5 to “review”

12. limit 5 to technical report

13. limit 5 to practice guideline

14. 6 or 7 or 8 or 9 or 10 or 11 or 12 or 13

15. limit 14 to English language

16. limit 15 to last 10 years

17. exp *marker/ or biological marker/ or clinical marker/

18. (marker* or biomarker* or factor* or classifier or signature* or target* or endpoint). ti, ab.

19. 17 or 18

20. 16 and 19

Reference

The Ovid strategy was conducted by following the guidance by BMA Library - MEDLINE Plus. Basic Course. Notes for OvidSP; 2012. http://www.google.co.uk/url?sa=t&rct=j&q=&esrc=s&frm=1&source=web&cd=1&ved=0ahUKEwjS7_OmodvJAhWGVhQKHZr0AZMQFggdMAA&url=http%3A%2F%2Fbma.org.uk%2F-%2Fmedia%2Ffiles%2Fpdfs%2Fabout%2520the%2520bma%2Flibrary%2Fmedline%2520plus%2520basic%2520course%2520manual%25202012.pdf&usg=AFQjCNGFxcWiS11CJsroeeIETAWjW0neUA
